# The parkin V380L variant is a genetic modifier of Machado–Joseph disease with impact on mitophagy

**DOI:** 10.1007/s00401-024-02762-6

**Published:** 2024-08-01

**Authors:** Jonasz J. Weber, Leah Czisch, Priscila Pereira Sena, Florian Fath, Chrisovalantou Huridou, Natasa Schwarz, Rana D. Incebacak Eltemur, Anna Würth, Daniel Weishäupl, Miriam Döcker, Gunnar Blumenstock, Sandra Martins, Jorge Sequeiros, Guy A. Rouleau, Laura Bannach Jardim, Maria-Luiza Saraiva-Pereira, Marcondes C. França, Carlos R. Gordon, Roy Zaltzman, Mario R. Cornejo-Olivas, Bart P. C. van de Warrenburg, Alexandra Durr, Alexis Brice, Peter Bauer, Thomas Klockgether, Ludger Schöls, Olaf Riess, The EUROSCA Network, Peter Bauer, José Berciano, Sylvia Boesch, Alexis Brice, Alexandra Durr, Sylvie Forlani, Paola Giunti, Heike Jacobi, Thomas Klockgether, Bela Melegh, Massimo Pandolfo, Olaf Riess, Tanja Schmitz-Hübsch, Ludger Schöls, Jörg B. Schulz, Giovanni Stevanin, Sandra Szymanski, Sophie Tezenas du Montcel, Dagmar Timmann, Bart P. C. van de Warrenburg, Thorsten Schmidt

**Affiliations:** 1 Institute of Medical Genetics and Applied Genomics, University of Tübingen, 72076 Tübingen, Germany; 2 Department of Human Genetics, Ruhr University Bochum, 44801 Bochum, Germany; 3 Department of Clinical Epidemiology and Applied Biometry, University of Tübingen, 72076 Tübingen, Germany; 4 i3S - Instituto de Investigação e Inovação em Saúde, Universidade do Porto, 4200-135 Porto, Portugal; 5 IPATIMUP - Institute of Molecular Pathology and Immunology, University of Porto, 4200-135 Porto, Portugal; 6 ICBAS School of Medicine and Biomedical Sciences, University of Porto, 4050-313 Porto, Portugal; 7 Department of Neurology and Neurosurgery and The Neuro (Montreal Neurological Institute‐Hospital), McGill University, Montréal, H3A 1A1 Canada; 8 Departamento de Medicina Interna, Faculdade de Medicina, Universidade Federal do Rio Grande do Sul, Porto Alegre, 90035-903 Brazil; 9 Serviço de Genética Médica, Hospital de Clínicas de Porto Alegre, Porto Alegre, 90035-903 Brazil; 10 Departamento de Bioquímica, Universidade Federal do Rio Grande do Sul, Porto Alegre, 90035-003 Brazil; 11 Universidade Estadual de Campinas (UNICAMP), Campinas, 13083-970 Brazil; 12 Department of Neurology, Tel Aviv University, 69978 Tel Aviv, Israel; 13 Neurogenetics Research Center, Instituto Nacional de Ciencias Neurológicas, 15003 Lima, Peru; 14 Neurogenetics Working Group, Universidad Científica del Sur, 15067 Lima, Peru; 15 Department of Neurology, Donders Institute for Brain, Cognition, and Behaviour, Radboud University Medical Center, 6525 Nijmegen, The Netherlands; 16 Department of Genetics and Cytogenetics, 4 AP-HP, Groupe Hospitalier Pitié-Salpêtrière, 75013 Paris, France; 17 Sorbonne Université, Institut du Cerveau - Paris Brain Institute - ICM, Inserm, CNRS, APHP, University Hospital Pitié-Salpêtrière, 75013 Paris, France; 18 Centogene GmbH, 18055 Rostock, Germany; 19 Clinic for Internal Medicine, Department of Hematology, Oncology, Palliative Medicine, University Medicine Rostock, 18057 Rostock, Germany; 20 German Center for Neurodegenerative Diseases (DZNE), 53127 Bonn, Germany; 21 Department of Neurology, University Hospital Bonn, 53127 Bonn, Germany; 22 Department of Neurology and Hertie-Institute for Clinical Brain Research, University of Tübingen, 72076 Tübingen, Germany; 23 German Center of Neurodegenerative Diseases (DZNE), 72076 Tübingen, Germany

**Keywords:** *PRKN*, Spinocerebellar ataxia type 3, SCA3, Polyglutamine disease, SNP, Aggregation

## Abstract

Machado–Joseph disease (MJD) is an autosomal dominant neurodegenerative spinocerebellar ataxia caused by a polyglutamine-coding CAG repeat expansion in the *ATXN3* gene. While the CAG length correlates negatively with the age at onset, it accounts for approximately 50% of its variability only. Despite larger efforts in identifying contributing genetic factors, candidate genes with a robust and plausible impact on the molecular pathogenesis of MJD are scarce. Therefore, we analysed missense single nucleotide polymorphism variants in the *PRKN* gene encoding the Parkinson's disease-associated E3 ubiquitin ligase parkin, which is a well-described interaction partner of the MJD protein ataxin-3, a deubiquitinase. By performing a correlation analysis in the to-date largest MJD cohort of more than 900 individuals, we identified the V380L variant as a relevant factor, decreasing the age at onset by 3 years in homozygous carriers. Functional analysis in an MJD cell model demonstrated that parkin V380L did not modulate soluble or aggregate levels of ataxin-3 but reduced the interaction of the two proteins. Moreover, the presence of parkin V380L interfered with the execution of mitophagy—the autophagic removal of surplus or damaged mitochondria—thereby compromising cell viability. In summary, we identified the V380L variant in parkin as a genetic modifier of MJD, with negative repercussions on its molecular pathogenesis and disease age at onset.

**Supplementary Information:**

The online version contains supplementary material available at 10.1007/s00401-024-02762-6.

## Introduction

Machado–Joseph disease (MJD) or spinocerebellar ataxia type 3 (SCA3) is one of nine known neurodegenerative polyglutamine (polyQ) diseases caused by an expansion of an exonic glutamine-coding CAG repeat tract within the respective disease-specific genes [22]. While precise data on MJD prevalence is lacking and complicated by both ethnic and geographic variations, this autosomal dominant disorder is considered the most common type worldwide amongst spinocerebellar ataxias, which exhibit an estimated global frequency of 3 in 100,000 individuals [43]. In MJD, the expanded tract lies in exon 10 of the *ATXN3* gene and translates into an elongated polyQ stretch at the C-terminus of the disease protein ataxin-3 [17, 43]. Physiologically, ataxin-3 acts as a deubiquitinating enzyme (DUB), which is defined by its N-terminal catalytic Josephin domain and up to three ubiquitin-interacting motifs (UIMs). It binds ubiquitinated proteins and trims their polyubiquitin chains, thereby modulating substrate degradation via proteasomal or autophagosomal machineries [6, 19, 24, 39, 56].

The polyQ-expansion of ataxin-3 results in its misfolding and aggregation as characteristic intraneuronal inclusions [35, 42]. These changes have been linked to disturbances of various cellular mechanisms, including DNA damage repair, mitochondrial function, protein quality control and turnover, and transcriptional regulation, eventually leading to neuronal dysfunction and primarily cerebellar degeneration in patients [26, 38, 52].

In *ATXN3*, the normal number of CAG triplets ranges from 12 to 44, while pathologically increased CAG stretches span from 56 to 87 repeats. Transitional lengths with incomplete penetrance lie in between [29, 34]. The age at onset (AAO) of MJD shows a wide range (between 5 and 75 years of age), with a statistical mean in the third to fourth decade of life, and correlates inversely with the number of CAG repeats [23, 38, 43, 48]. However, the number of CAG repeats explains only about 50% of the variability in AAO, suggesting the influence of other genetic or environmental factors [23, 50].

While certain single nucleotide polymorphisms (SNPs) in genes, such as *FAN1*, *RAG1/2*, or *TRIM29*, the presence of the *APOE-ε2* allele, as well as non-pathologically expanded CAG repeat loci in *ATXN2*, *ATN1* and *HTT* have already been demonstrated as modifiers of the AAO and pathology in MJD [2, 5, 30, 37, 47], the influence of variants in known physical interactors and their impact on the pathophysiology remain underinvestigated. The E3 ubiquitin ligase parkin, whose mutations are notoriously associated with juvenile manifestations of Parkinson’s disease [18, 58], is one of the prime binding partners of ataxin-3, linked to its role within the ubiquitin system [1, 11, 12, 55]. Both proteins interact in a bimodal fashion, primarily based on the binding of parkin’s ubiquitin-like (Ubl) domain with ubiquitin-interacting motifs (UIM) of ataxin-3, and of the in-between-ring domain (IBR)/really-interesting-new-gene domain 2 (RING2) region of parkin with ataxin-3’s Josephin domain, which is unaffected by the polyQ expansion [12]. One central function of parkin is its involvement in mitophagy, an autophagy-based mechanism for the removal of damaged or excess mitochondria [33], which may represent a potential site of interaction with ataxin-3 [16, 45]. Interestingly, an earlier study showed that polyQ-expanded ataxin-3 may contribute to an undesirably enhanced removal of parkin via autophagy, thereby contributing to the molecular pathogenesis of MJD [12].

We selected the three most common missense SNP variants in the parkin-coding gene *PRKN*, namely rs1801474 (gnomAD ID: 6-162201165-C-T; exon 4, c.601G > A; S167N), rs1801582 (gnomAD ID: 6-161386823-C-G; exon 10, c.1239G > C; V380L), and rs1801334 (gnomAD ID: 6-161360193-C-T; exon 11, c.1281G > A; D394N) (all gnomAD IDs referring to the GRCh38/hg38 genome build; gnomAD v4.1.0), with potential impact on its functional interaction with ataxin-3. Using genotype-AAO correlation analysis in a large cohort of patients combined with cell-based functional investigations, we examined the contribution of selected *PRKN* missense variants to the molecular pathogenesis of MJD.

## Materials and methods

### DNA samples of MJD patients

An overview of the MJD patient cohorts from which DNA samples were obtained and their detailed specifications can be found in Supplementary Table S1.

### PCR

The amplification was carried out with standard PCR conditions using a G-Storm thermal cycler (AlphaMetrix Biotech GmbH, Rödermark, Germany). The complete list of employed primers can be found in Supplementary Table S2. The reaction mixtures and thermal cycler programmes for each SNP are provided in Supplementary Table S3 and S4, respectively.

### High-resolution melting analysis

Genotyping of selected SNPs in the *PRKN* gene was performed by high-resolution melting analysis carried out on a LightCycler 480 (Roche Diagnostics, Mannheim, Germany) using the LightCycler 480 High Resolution Melting Master Kit (Roche Diagnostics) following the manufacturer's instructions. For a reliable detection of genotype-specific melting peaks, an unlabelled probe was added. To ensure a sufficient amplification of DNA strands complementary to the probe, an asymmetric PCR with a ratio of reverse primer to forward primer of 10:1 was performed. The complete list of employed primers and probes can be found in Supplementary Table S2. Alternatively, genotyping was performed via TaqMan. The reaction conditions for each SNP can be found in the Supplementary Table S5.

### Sanger sequencing

Results from the high-resolution melting analysis were validated by Sanger sequencing. After PCR amplification, samples were purified using the QIAquick PCR Purification Kit (QIAGEN, Hilden, Germany), according to the manufacturer's instructions. Sequencing reactions were carried out on the CEQ 8000 Genetic Analysis System Sequencer (Beckman/AB SCIEX, Krefeld, Germany), following instructions of the GenomeLab Dye Terminator Cycle Sequencing with Quick Start Kit Manual (Beckman Coulter). Supplementary Table S2 provides a comprehensive list of employed sequencing primers.

### Expression constructs

For V5-, Xpress-, or GFP-tagged ataxin-3 overexpression, pcDNA3.1 (Thermo Fisher Scientific, Waltham, MA USA) and pEGFP-N2 (Clontech, Mountain View, CA, US) vectors encoding canonical isoform 2 of ataxin-3 (UniProt ID: P54252-2; ataxin-3c) with 15, 77, or 148 glutamines (15Q, 77Q, 148Q) were utilised. Overexpression of parkin (UniProt ID: O60260-1) was achieved using pcDNA3.1 vectors encoding wild-type parkin (parkin WT) and parkin carrying an Val380Leu amino acid exchange (parkin V380L), both N-terminally fused to a 6xMyc tag. The Val380Leu exchange was achieved by site-directed mutagenesis using forward primer GGAGTGCAGTGCCCTATTTGAAGCCTC and reverse primer GAGGCTTCAAATAGGGCACTGCACTCC. Correct integration of mutations was confirmed by Sanger sequencing using forward primer CTGCCGGGAATGTAAAGAAG. For Tet-off system-based expression of parkin WT or V380L, the respective cDNA was cloned into a pTRE responder vector, and used in combination with a pTET-RCA2 plasmid as described earlier [55]. For microscopically visualising mitochondria, a mammalian expression vector coding for the fluorescent protein DsRed2 and fused to a mitochondrial targeting signal (pDsRed2-Mito; Takara Bio USA, Inc., San Jose CA, US) was employed.

### Cell culture

For cell culture experiments, HEK293T wild-type (293T WT) cells (ATCC: CRL-11268) and HEK293T *ATXN3* knockout (293T *ATXN3*^*−/−*^) cells [55] were cultured in Dulbecco’s modified Eagle’s medium (DMEM) supplemented with 10% foetal bovine serum (FBS), 1% non-essential amino acids (MEM NEAA), and 1% Antibiotic–Antimycotic (A/A) (all Gibco, Thermo Fisher Scientific) in 5% CO_2_ at 37 °C. Transient cell transfection was conducted for 72 h using Attractene (QIAGEN) or Turbofectin 8.0 (OriGene Technologies, Inc., Rockville, MD, US) reagents according to the manufacturers’ protocols, with an approximate transfection efficiency of 50% transfected cells as assessed using a representative pEGFP-N2 reporter construct and fluorescence microscopy. For depolarization of mitochondria, cells were treated with various concentrations of carbonyl cyanide m-chlorophenylhydrazone (CCCP; Merck, Darmstadt, Germany) for 24 h. For inhibition of proteasomal degradation, cells were incubated with 2.5 µM MG132 (Merck) or 0.5 µM epoxomicin (Selleck Chemicals, Cologne, Germany) for 24 h. For inhibiting autophagy, 25 nM bafilomycin A1 (Selleck Chemicals) was administered. Dimethyl sulphoxide (DMSO) was used as vehicle control.

### Protein stability analysis

Analysis of protein stability was performed using a Tet-off system as previously described [55]. In brief, 293T *ATXN3*^*−/−*^ cells were transfected with pTRE-parkin WT or V380L responder constructs and a pTET-RCA2 vector in a 1:1 ratio, and, if desired, additionally in combination with a pcDNA3.1 Xpress-Atx3 148Q plasmid. Expression was terminated by the addition of doxycycline (Merck; 4.5 µM) at desired time points before cell harvest.

### Cell viability assay

Assessment of cell viability was performed as previously described [36] with the following specifications: 5,000 cells/well were seeded in 96-well cell culture plates (ViewPlate-96 Black, Perkin Elmer, Massachusetts, USA) and transfected 24 h later using Turbofectin 8.0 for 72 h. Cells were treated with 12.5 µM CCCP or vehicle control DMSO for the last 24 h. Afterwards, culture medium was aspirated, cells incubated in fresh medium containing the resazurin-based PrestoBlue™ Cell Viability Reagent (Thermo Fisher Scientific) in a 1:10 ratio under standard culture conditions for 60 min. Fluorescence signals were measured at 535 nm (excitation)/615 nm (emission) using a Synergy HT plate reader and the Gen5 software (both BioTek Instruments, Winooski, VT, USA).

### Fluorescence-activated cell sorting (FACS) analysis

Cell death was measured using fluorescence-activated cell sorting (FACS) analysis as previously described [41]. Briefly, transfected and treated 293T WT cells were trypsinised, pelleted, washed with 1× DBPS and stained with 2.5% (v/v) of 7-aminoactinomycin D (7-AAD) (BD Biosciences, San Jose, CA, USA) in CliniMACS^®^ PBS/EDTA buffer (Miltenyi Biotec, Bergisch Gladbach, Germany) at room temperature for 3–5 min. Afterwards, cells were analysed using a BD LSRFortessa™ X-20 cytofluorometer with the BD FACSDiva™ v9.0 software (both BD Biosciences). The flow cytometry results were further analysed using FlowJo™ v10.10 Software (FlowJo LLC, BD Life Sciences, Ashland, OR, USA).

### Immunofluorescence staining and microscopy

Immunofluorescence staining of cells was performed as previously described [53]. In brief, 5000 293T cells per well were seeded on an 8-well chamber slide (80841, Ibidi, Gräfelfing, Germany), treated as desired, and subjected to fixation in 4% (w/v) paraformaldehyde in 1× Dulbecco’s phosphate-buffered saline (DPBS), followed by a 1-h permeabilization and fixation step in 10% (w/v) bovine serum albumin, 0.5% (v/v) Triton X-100, and 0.02% (w/v) NaN_3_ in 1× DPBS. Cells were incubated with primary antibodies mouse anti-Myc-tag (1:500; clone 9B11, #3739, Cell Signaling, Danvers, MA, US) or goat mouse anti-parkin (1:1,000; clone PRK8, MAB5512, Merck) at 4 °C overnight. The next day, cells were washed and incubated with goat anti-mouse IgG Alexa Fluor 555 (1:500; A-21424, Thermo Fisher Scientific) or goat anti-mouse IgG Alexa Fluor Plus 594 (1:500; A32742, Thermo Fisher Scientific) secondary antibodies at room temperature for 1 h. After washing, cells were mounted with VECTASHIELD® Antifade Mounting Medium with DAPI (H-1200, Vector Laboratories, Newark, CA, US) using coverslips and sealed with transparent nail polish.

Epi-fluorescence images were taken at a 200×, 400×, and 630× magnification on an Axioplan 2 imaging microscope equipped with an ApoTome, Plan-Neofluar 20 × /0.50, Plan-Neofluar 40×/0.75, and Plan-Neofluar 63×/1.4 Oil objectives, and an AxioCam MRm camera, using the AxioVision 4.3 imaging software (all Zeiss, Oberkochen, Germany).

Confocal fluorescence images were acquired at a 1000× magnification on an ECLIPSE Ti2 microscope equipped with an CSU-W1 SoRa confocal system and an CFI SR HP Plan Apochromat Lambda S 100 × C Sil objective, using NIS-Elements AR 5.42 (all Nikon, Tokio, Japan).

Morphological assessment of GFP-Atx3 148Q aggregates was performed using the Fiji software [40], and an in-house established macro for semi-automated analysis to measure cross-sectional area and roundness of detected GFP-positive particles. Based on an equivalent circular area (ECA) to the assessed aggregate area (*A*_ag_), a corresponding aggregate diameter was extrapolated (*d*_ECA_) [21], using the following equation: ${d}_{\text{ECA}} =\sqrt{4\times \frac{{A}_{\text{ag}}}{\pi }}.$

### Protein extraction

For protein extraction, 293T cells were dissociated by gentle pipetting and transferred to 2.0 mL tubes. Cell pellets were obtained by centrifugation at 500×*g* for 5 min followed by aspiration of the supernatant. Pellets were washed once with cold 1× DPBS. Homogenization was conducted by resuspending the cell pellet in RIPA buffer (50 mM Tris pH 7.5, 150 mM NaCl, 0.1% SDS, 0.5% sodium deoxycholate and 1% Triton X-100) containing cOmplete™ protease inhibitor cocktail (Roche Diagnostics) and ultrasonication using a Sonopuls ultrasonic homogenizer (Bandelin electronic, Berlin, Germany) for 3 s and 10% pulse duration at 10% power. Homogenates were mixed with glycerol to a final concentration of 10%. For cell lysate preparation, protein extracts were incubated for 15 min on ice followed by a 15-min centrifugation at 4 °C and 16,100×*g*. Supernatants were transferred to a fresh pre-cooled tube with addition of 10% glycerol. Protein concentrations were measured spectrophotometrically in a microtiter plate using Bradford reagent (Bio-Rad Laboratories, Basel, Switzerland). Samples were stored at -80 °C until further analysis.

### Immunoprecipitation

GFP-tagged proteins were immunoprecipitated using GFP-Trap agarose according to the manufacturer’s protocol (ChromoTek and Proteintech Germany, Planegg-Martinsried, Germany) with the following specifications: For immunoprecipitation (IP), 1000 µg of total protein was incubated with 20 µL of agarose bead slurry in a 1.5 mL reaction tube, rotating end-over-end at 4 °C and 10 rpm for 1 h. After IP, samples were eluted in 80 µL of 4× LDS sample buffer (1 M Tris pH 8.5, 50% (v/v) glycerol, 8% (w/v) LDS, 2 mM EDTA, 0.1% (w/v) Orange G) mixed with Trap dilution buffer (10 mM Tris, pH 7.5, 150 mM NaCl, 0.5 mM EDTA) in a ratio 1:1, supplemented with 100 mM dithiothreitol (DTT) and heat-denatured at 70 °C and 600 rpm for 10 min. Samples were subsequently analysed by western blotting.

### Western blotting

Western blotting was performed according to standard procedures. Briefly, protein extracts were mixed with a 4× LDS sample buffer in a ratio 3:1 and supplemented with 100 mM DTT. After heat-denaturing for 10 min at 70 °C, protein samples were electrophoretically separated using custom-made Bis–Tris gels and MOPS electrophoresis buffer (50 mM MOPS, 50 mM Tris pH 7.7, 0.1% SDS, 1 mM EDTA). Proteins were transferred on Amersham™ Protran™ Premium 0.2 µm nitrocellulose membranes (Cytiva, Freiburg, Germany) using Bicine/Bis–Tris transfer buffer (25 mM Bicine, 25 mM Bis–Tris pH 7.2, 1 mM EDTA, 15% methanol) and a TE22 Transfer Tank (Hoefer, Inc., Holliston, MA, US) at 80 V and a maximum of 250 mA for 2 h.

After transfer, membranes were blocked for 45 min with 5% skim milk powder (Merck) in 1× TBS (10 mM Tris, pH 7.5, 150 mM NaCl) at room temperature, and probed overnight at 4 °C with primary antibodies diluted in TBS-T (TBS with 0.1% Tween 20). A detailed listing of applied primary antibodies can be found in Supplementary Table S6. Afterwards, membranes were washed with 1 × TBS-T and incubated at room temperature for 1 h with the respective secondary IRDye^®^ antibodies goat anti-mouse 680LT (P/N 926-68020), goat anti-mouse 800CW (P/N 926-32210), and goat anti-rabbit 800CW (P/N 926-32211) (all 1:10,000; LI-COR Biosciences, Lincoln, NE, US). After final washing with 1× TBS-T, fluorescence signals were detected using the LI-COR ODYSSEY^®^ FC and quantified with Image Studio 4.0 software (both LI-COR Biosciences).

### Filter retardation assay

Detection of SDS-insoluble ataxin-3 was performed as previously described [54]. Briefly, 1 µg of cell homogenates was diluted in 1× DPBS containing 2% SDS and 50 mM DTT. Afterwards, samples were heat-denatured at 95 °C for 5 min and filtered through an Amersham™ Protran™ 0.45 µm nitrocellulose membrane (Cytiva) using a Minifold^®^ II Slot Blot System (Schleicher & Schuell, Dassel, Germany). After blocking in 5% skim milk powder (Merck) in 1× TBS, membranes were incubated with respective primary and secondary antibodies. Fluorescence signals were detected using the LI-COR ODYSSEY^®^ FC and quantified with Image Studio 4.0 software (both LI-COR Biosciences).

### Statistical analysis

The statistical analysis to test the influence of rs1801582 in *PRKN* on the AAO of MJD patients was performed using the JMP software (JMP, Cary, NC, US; version 16.2.0). A *P*-value of ≤ 0.05 was considered statistically significant. The Hardy–Weinberg distribution of the polymorphism’s allele frequencies was verified using a chi-square (*χ*^2^) test. Non-evaluable samples were excluded from the initial cohort of MJD patients after the following criteria: no reliable genotyping result or missing values for CAG repeats or AAO. To exclude potential differences in the geographical distribution of rs1801582 genotypes in the three analysed MJD patient sub-cohorts, a Fisher–Freeman–Halton exact test was applied. To account for related samples, a family factor was applied. In the statistical analysis, the weighting of each family member was divided by the total number of family members. Thereby, each whole family was considered as one independent sample only. Testing for statistical significance between the distribution of CAG repeat length and AAO for each genotype was performed using a two-tailed Kruskal–Wallis test, followed by a Steel–Dwass all pairs analysis. To analyse the effect of the polymorphism in consideration with the already known effect of the CAG repeat length on the AAO, a multivariate linear regression analysis model was used with AAO as dependent and polymorphism and expanded CAG repeats as independent variables. The combined effect of CAG repeat length and polymorphism in additive and interactive models were compared with the effect of the CAG repeat length alone, using the coefficient of determination (*r*^2^) and the effect test function. The mean AAO for each genotype was adjusted to the mean CAG repeat length in the expanded allele and compared between the groups using the least square means adjustment tool of JMP. Linear equations for the AAO in dependence on the CAG repeat length were established for each genotype in prediction models, allowing a more accurate prediction of the AAO. Violin plots of respective datasets were generated with GraphPad Prism 10.1.1 (GraphPad Software, Dotmatics, Boston, MA, USA).

Data from the functional analysis were statistically analysed using GraphPad Prism 10.1.1 (GraphPad Software). The results are presented as bar charts with bars representing mean + standard error of the mean (s.e.m.) or violin plots featuring medians as well as the 25% and 75% percentiles, respectively. Statistical outliers were determined using the ROUT method with a default *Q* = 1%. One-sample *t*-test, Student's *t*-test, or one-way ANOVA with the respective post hoc analysis was applied. Significance was assumed with a *P*-value ≤ 0.05. For further details, see the respective figure legends.

## Results

### SNP rs1801582 in exon 10 in the *PRKN* gene fulfils criteria for subsequent correlation analysis

**Fig. 1:**
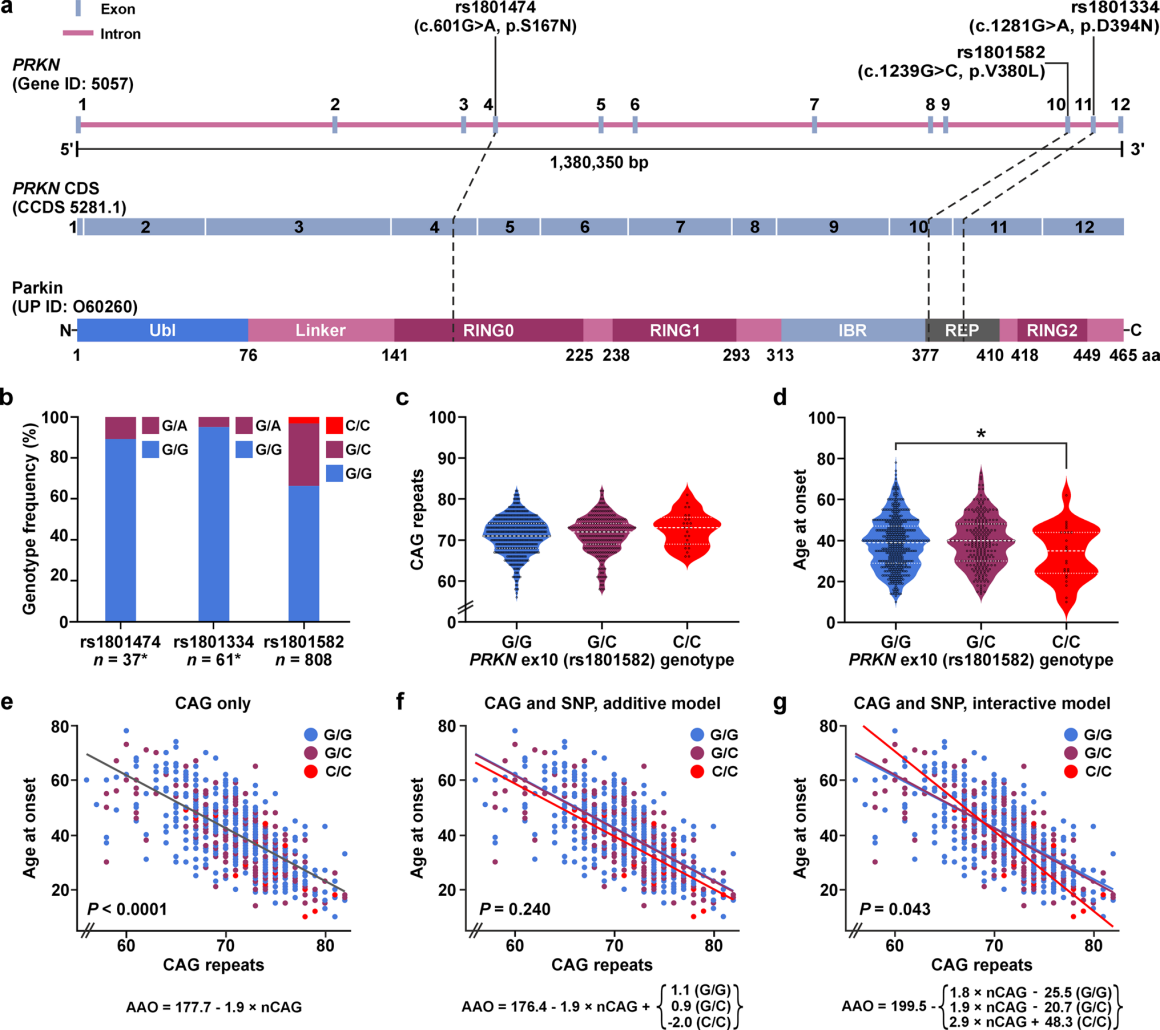
Characterization of the SNP rs1801582 in exon 10 of the *PRKN* gene in MJD patients and the influence of the different genotypes on the AAO. **a** The *PRKN* gene contains 12 exons and 11 introns. Among others, three SNPs within its coding region lead to amino acid changes in the encoded parkin protein: the G/A polymorphism rs1801474 in exon 4 leads to a serine to asparagine (S167N) change in the really-interesting-new-gene domain 0 (RING0) domain, rs1801582 (G/C) in exon 10 causes a valine to leucine (V380L) change in a region between the in-between-ring (IBR)-domain and the repressor element of parkin (REP); and rs1801334 (G/A) in exon 11 leads to an aspartic acid to asparagine (D394N) change in the repressor element. Ubl, ubiquitin-like domain. **b** Determination of genotype frequencies in a small pilot cohort of European MJD patients (sample numbers marked with an *) confirmed the expectedly low minor allele frequency for both rs1801474 in *PRKN* exon 4 and rs1801334 in *PRKN* exon 11. Analysis of the entire MJD cohort of 808 evaluable patient samples revealed robust frequencies for all genotypes of rs1801582 in *PRKN* exon 10. **c** Violin plots illustrate distribution of CAG repeat numbers per *PRKN* SNP rs1801582 genotype in 808 analysed MJD patients. No significant difference in the distributions was detected (*P* = 0.212). Dashed white lines indicate the median, and dotted white lines the 25% and 75% percentile, respectively. **d** Violin plots show the distribution of age at onset (AAO) per *PRKN* SNP rs1801582 genotype in 808 analysed MJD patients. A significant genotype effect (*P* = 0.035) was detected, with the AAO being significantly lower in the genotype C/C compared to G/G (*P = 0.040). After applying a family factor to account for related samples, a two-tailed Kruskal–Wallis test was performed, followed by a Steel–Dwass all pairs analysis for pair-wise comparison. Dashed white lines indicate the median, and dotted white lines the 25% and 75% percentile, respectively. **e** Multivariate linear regression analysis. The expected inverse correlation between CAG repeats and AAO was confirmed (*P* < 0.0001). **f** In an additive model of multivariate linear regression, the AAO for genotype C/C was approximately 3 years earlier than for the other two genotypes. However, the effect of the polymorphism alone did not reach statistical significance (*P* = 0.240). **g** In an interactive model of multivariate linear regression, a higher CAG repeat length in combination with the C/C genotype had a stronger impact on the AAO in patients, with the interaction between the polymorphism and the CAG repeat length being statistically significant (*P* = 0.043)

The *PRKN* gene contains hundreds of variants in its coding region of which the three most frequent missense SNPs are rs1801474 in exon 4 (c.601G > A; p.S167N), rs1801582 in exon 10 (c.1239G > C; p.V380L) and rs1801334 in exon 11 (c.1281G > A; p.D394N) (Fig. 1a), with minor allele frequencies of 0.0379, 0.1684, and 0.0341, respectively (gnomad.broadinstitute.org/gene/ENSG00000185345; GRCh38/hg38 genome build; gnomAD v4.1.0). While the minor allele frequencies for SNPs rs1801474 and rs1801334 were low in comparison to rs1801582, we still included these variants in our genotyping approach for confirmatory purposes. For SNP genotyping, we established high-resolution melting assays using unlabelled probes specific for all three SNPs (Supplementary Fig. S1a-c), and results were validated by Sanger sequencing. Following the expectations, our pilot analyses in a smaller cohort of randomly selected MJD patient samples confirmed that the minor allele frequencies for SNPs rs1801474 and rs1801334 were too low for robust statistics (Fig. 1b), reaching values of 0.0541 and 0.0246, respectively. We therefore restricted our study to SNP rs1801582 and analysed all 911 MJD patient samples collected within the EUROSCA, EuSAge, and Montreal cohorts (Supplementary Table S1). After quality control, a total of 808 evaluable samples remained, which showed frequencies of 66.3% for genotype G/G, 30.6% for genotype G/C, and 3.1% for the genotype C/C for rs1801582 (Fig. 1b), with a calculated minor allele frequency of 0.1838, featuring a statistically even geographic distribution of all genotypes (*P* = 0.109) across the three analysed sub-cohorts (Supplementary Table S1; Supplementary Fig. S1d). Moreover, we compared SNP rs1801582 genotype distributions in the EUROSCA, EuSAge, and Montreal sub-cohorts with ancestry-specific general populations and detected no significant deviation between observed and expected distributions (Supplementary Table S7).

### SNP rs1801582 in *PRKN* lowers the AAO of MJD patients

To investigate the influence of SNP rs1801582 in *PRKN* exon 10 on the AAO in MJD patients, statistical analyses were performed, which included the integration of a family factor to correct for the influence of related samples in spite of the lack of further pedigree information. First, we tested whether the number of CAG repeats differed between the three genotype groups across the individual sub-cohorts, as this could bias potential effects on the AAO. We did not find any significant deviation in the number of CAG repeats (Supplementary Table S8). When testing for differences in the AAO between the genotype groups in the EUROSCA, EuSAge, and Montreal cohort individually, no significant effects of the rs1801582 genotype were detected (Supplementary Table S8).

As this lack of effects might be attributed to the relatively small number of MJD patients per cohort and the minor allele frequency of rs1801582, we decided to extend our analysis to a combination of all three sub-cohorts. While we again did not observe any differences in the CAG repeat numbers between the genotype groups in the combined cohort (*P* = 0.213) (Table 1; Fig. 1c), we found a significantly earlier AAO in MJD patients with the C/C genotype, showing a difference of about 6 years compared to patients with the G/G or G/C genotype (*P* = 0.035) (Table 1; Fig. 1d). To account for slight differences in the CAG repeat number between the different genotype groups, the mean AAO was adjusted. Taking the higher CAG repeat number statistically into consideration, the AAO of patients with the C/C genotype was about 3 years earlier compared to patients with a G/G or G/C genotype (Table 1).

**Table 1:** Mean unadjusted and adjusted age at onset per *PRKN* SNP rs1801582 genotype, and analysis of differences in CAG repeat numbers and age at onset between genotypes in the combined MJD cohort

Genotype	All combined	G/G	G/C	C/C	Kruskal–Wallis test, *P*
Patients, *n*	808	536	247	25	
CAG repeats, *n* [range]	71.2 ± 0.2 [56–82]	71.1 ± 0.2 [56–82]	71.4 ± 0.3 [58–82]	72.7 ± 0.9 [66–81]	0.213
AAO, years [range]	39.9 ± 0.4 [10–78]	40.5 ± 0.5 [10–78]	39.4 ± 0.8 [14–73]	33.7 ± 2.6 [10–62]	**0.035**
AAO adjusted, years	n/a	40.6 ± 0.4	40.3 ± 0.6	37.7 ± 1.9	n/a

The age at onset (AAO) was adjusted to the mean number of CAG repeats of the combined MJD cohort. Differences in the distribution of CAG repeat lengths and AAO between genotypes were analysed using a two-tailed Kruskal–Wallis test. Values for CAG numbers and AAO are presented as means ± s.e.m including the range. Significant *P*-values are highlighted in bold

The CAG repeat length in *ATXN3* is the prime determinant of the AAO of MJD patients. As expected, we observed a highly significant inverse correlation between CAG repeat numbers and the AAO in a multivariate linear regression analysis (*P* < 0.0001), both for the individual sub-cohorts (Supplementary Table S9) and combined cohorts of MJD patients (Fig. 1e; Table 2; Supplementary Table S9). Taking the CAG numbers into account, we then evaluated whether the SNP rs1801582 in *PRKN* had an additional impact on the AAO. For this, we used an additive regression model including the AAO as the dependent variable, and CAG repeats and SNP genotype as independent variables, and analysed the EUROSCA, EuSAge, and Montreal sub-cohorts (Supplementary Fig. S2a, c, e; Supplementary Table S9). While there was a trend towards an earlier AAO in MJD patients carrying the C/C genotype, the analysis did not show statistical significance. Therefore, we applied an alternative model, considering a potential biological interrelationship between the CAG repeat number in *ATXN3* and the genotype on SNP rs1801582 in *PRKN.* Using this interactive model, an impact of the rs1801582 C/C genotype became more apparent, with a trend towards an earlier AAO in patients with higher CAG repeat numbers (Supplementary Fig. S2b, d, f; Supplementary Table S9).

**Table 2:** Multivariate linear regression analysis with additive and interactive models shows a significant effect of the *PRKN* SNP rs1801582 in interaction with the *ATXN3* CAG repeats in the combined MJD cohort

CAG repeats		CAG repeats and SNP, additive model			CAG repeats and SNP, interactive model		
*r*^2^	*P* (CAG)	*r*^2^	Δ*r*^2^	*P* (SNP)	*r*^2^	Δ*r*^2^	*P* (CAG*SNP)
0.497	**< 0.0001**	0.498	0.001	0.240	0.503	0.006	**0.043**

Models with the influence of the *ATXN3* CAG repeats alone, as well as the influence with an inclusion of the *PRKN* SNP rs1801582 in an additive and an interactive model were examined. CAG repeats had an expected inverse correlation with the age at onset (AAO) in the combined cohort of MJD patients. In an interactive model, *r*^2^ increased with the addition of the SNP. The effect of the polymorphism in interaction with the CAG repeats had a significant impact on the AAO, in contrast to the sole influence of the SNP, which did not reach statistical significance. *r*^2^, coefficient of determination, indicates the amount of variation in the dependent variable, predictable from the independent variable. Significant *P*-values are highlighted in bold

Corresponding to our previous analysis, we decided to combine cohorts and repeated the multivariate linear regression analyses using the additive and interactive models (Fig. 1e–g; Supplementary Figure S4; Table 2; Supplementary Table S9). Using this approach, we statistically consolidated the previously detected trends in the interactive model, observing significant effects for both the combination of EUROSCA and EuSAge sub-cohorts (*P* = 0.044) (Supplementary Fig. S4b; Supplementary Table S9) and the combination of all sub-cohorts (*P* = 0.043) (Fig. S1g; Table 2). Noteworthily, based on the linear equation of our additive model (Fig. 1f), the rs1801582 C/C genotype lowers the AAO in MJD patients by about 3 years, which is consistent with our previous estimations.

However, when performing a sensitivity analysis by replacing the generally applied family factor with a random selection of one representative sample per analysed MJD family in the combined cohort, the statistically significant correlation between the rs1801582 genotype and AAO was lost, while the overall trends remained (Supplementary Fig. S4). This might be attributed to the impact of a stochastically driven varying genotype distribution. Nevertheless, the generally persisting tendencies of an earlier AAO in MJD patients carrying the C/C genotype for rs1801582 in *PRKN* exon 10, along with the significant results using the family factor-adjusted analysis of the combined cohort, gave us confidence to explore the functional ramifications of the variant in *PRKN* on the molecular pathology of MJD.

### Parkin V380L does not alter soluble or aggregate levels of ataxin-3 but weakens the protein–protein interaction of both proteins

**Fig. 2:**
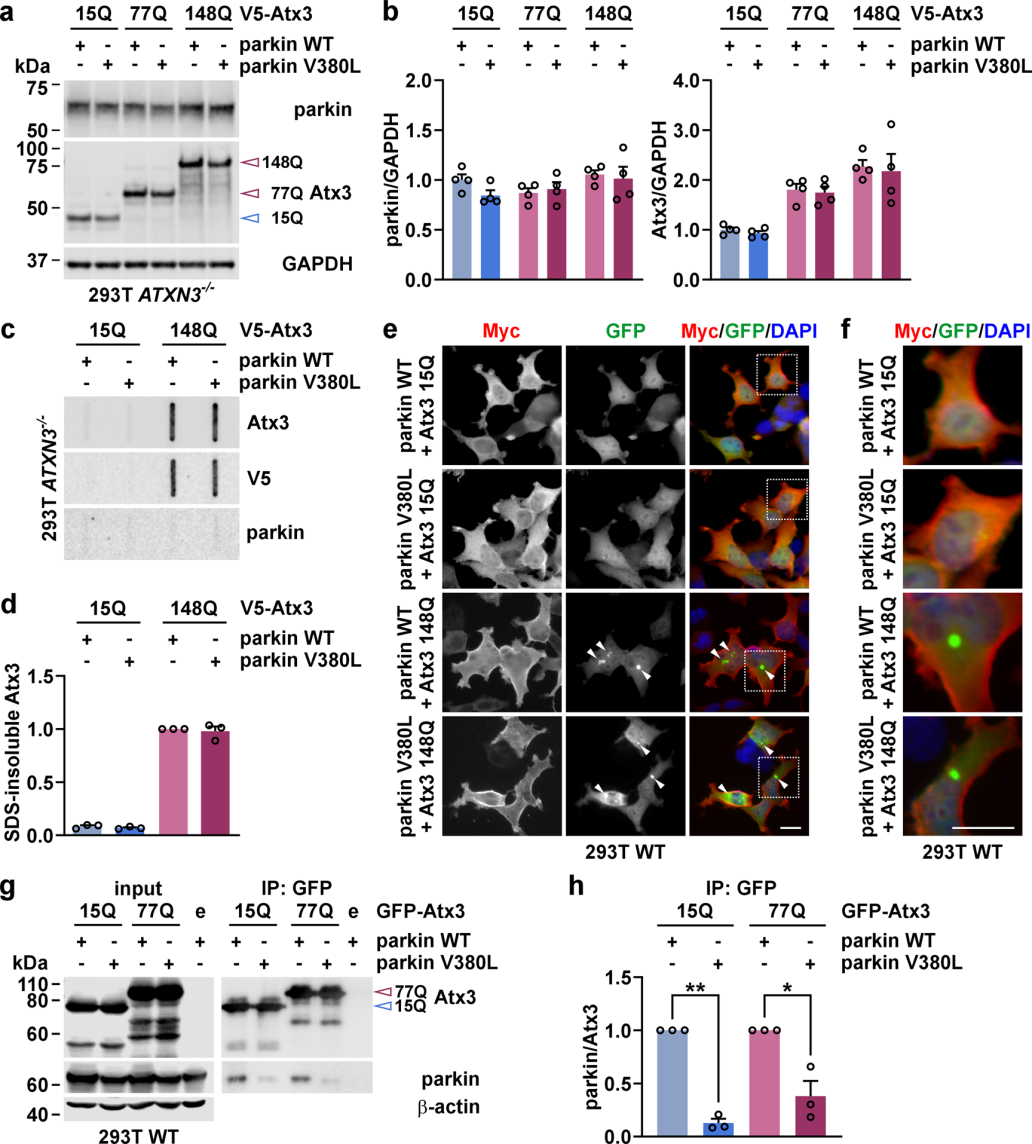
Parkin V380L does not influence soluble or insoluble ataxin-3 levels but reduces ataxin-3 binding. **a** Western blotting of 293T *ATXN3*^−/−^ cells co-expressing V5-Atx3 15Q, 77Q, or 148Q and 6xMyc-parkin WT or V380L. Membranes were detected with antibodies against respective proteins, revealing that parkin variants do not affect ataxin-3 protein levels and vice versa. GAPDH served as loading control. **b** Densitometric quantification of parkin and ataxin-3 levels, normalised to GAPDH. All values were additionally normalised to the mean of the control group Atx3 15Q/parkin WT. *n* = 4. Bars represent mean + s.e.m. **c** Filter retardation analysis of 293T *ATXN3*^−/−^ cells expressing parkin WT or V380L in combination with V5-Atx3 15Q and 148Q. SDS-insoluble protein species were detected using ataxin-3- and V5-tag-specific antibodies. Membranes were additionally stained with an antibody against parkin. Ataxin-3 aggregate levels remain unaltered upon overexpression of parkin variants. **d** Densitometric quantification of SDS-insoluble ataxin-3. Within each experimental replicate, values were additionally normalized to the Atx3 148Q/parkin WT control. *n* = 3. Bars represent mean + s.e.m. **e** Epi-fluorescence microscopy of 293T WT cells, co-transfected with 6xMyc-parkin WT or V380L and GFP-Atx3 15Q or 148Q. Parkin was detected using a Myc tag-specific antibody (red), while ataxin-3 was visualised via its GFP tag (green). DAPI was used as a nuclear counterstain (blue). Parkin variants do not alter the appearance of ataxin-3 aggregates (indicated by white arrowheads) and show no colocalization with them. **f** Magnifications of areas marked with white dashed boxes in **e**. **g** Western blotting of immunoprecipitated GFP-Atx3 15Q or 148Q co-expressed with 6xMyc-tagged parkin WT and V380L in 293T WT cells using a GFP-Trap approach. Immunoprecipitated GFP-Atx3 and co-precipitated parkin were detected using target-specific antibodies. Co-immunoprecipitation analysis shows reduced binding of parkin V380L with both wild-type and polyQ-expanded ataxin-3. β-actin served as loading control. **h** Densitometric quantification of co-precipitated parkin. Within each experimental replicate, values were normalised to Atx3 15Q/parkin WT or Atx3 70Q/parkin WT. *n* = 3. Bars represent mean + s.e.m. One sample *t*-test (comparisons to the respective control group Atx3 15Q/parkin WT or Atx3 70Q/parkin WT); *P ≤ 0.05; **P ≤ 0.01. Blue-rimmed arrowhead indicates ataxin-3 15Q and purple-rimmed arrowhead shows ataxin-3 77Q or 148Q. e = GFP empty vector. Scale bars = 20 µm

The SNP rs1801582 (c.1239G > C) within the *PRKN* gene leads to a valine to leucine amino acid exchange at position 380 (V380L) in the encoded parkin protein. To assess the functional consequences of the amino acid variant within parkin on the molecular pathogenesis of MJD, that may explain the observed decrease in the AAO of patients, we generated N-terminally 6xMyc-tagged parkin constructs with and without the respectively encoded V380L variant (for a schematic representation, see Supplementary Fig. S5a). Overexpression of parkin WT and V380L in 293T WT cells showed both comparable soluble protein levels as assessed by western blotting (Supplementary Fig. S5b). A similar intracellular distribution of both variants was confirmed by epi-fluorescence microscopy (Supplementary Fig. S5c, d) and ascertained by testing the specificity of the applied immunostaining (Supplementary Fig. S6). Moreover, protein stability analysis did not detect any difference between parkin WT and V380L, neither when expressed alone (Supplementary Fig. S7a, b) nor in combination with polyQ-expanded (148Q) ataxin-3 (Supplementary Fig. S7c, d). As earlier studies suggested an involvement of parkin in the elimination of polyglutamine-expanded disease proteins via its function as an E3 ubiquitin ligase and improvement of proteasomal function [49], we sought to investigate potential effects of the variant on soluble and insoluble levels of wild-type and polyQ-expanded proteins. For this, we overexpressed 6xMyc-parkin WT or V380L in combination with V5-tagged wild-type (15Q) or polyQ-expanded (77Q or 148Q) ataxin-3 (Atx3) in 293T *ATXN3*^−/−^ cells, and performed western blotting of soluble ataxin-3 levels (Fig. 2a) as well as filter retardation analysis of SDS-insoluble ataxin-3 species (Fig. 2c). Neither ataxin-3 nor parkin showed a mutual influence of their soluble protein levels, regardless of their polyQ length or the V380L variant in the respective protein (Fig. 2a, b). Moreover, we did not observe any differences between both parkin variants on aggregate levels of V5-Atx3 148Q (Fig. 2c, d), which we confirmed using an alternative GFP-tagged ataxin-3 overexpression construct (Supplementary Fig. S8a, b). To assess possible consequences on morphological features of aggregates formed by GFP-tagged ataxin-3, we analysed their cross-sectional area, equivalent circular area diameter and roundness using epi-fluorescence microscopy. None of these three analysed parameters showed alterations induced by the presence of the parkin variants (Supplementary Fig. S9a, b). Importantly, parkin was also not present in trapped SDS-insoluble polyQ-expanded ataxin-3 (Fig. 2c; Supplementary Fig. S8a). Noteworthily, we did not detect colocalization of parkin with ataxin-3 aggregates using epi-fluorescence microscopy of 293T cells expressing GFP-Atx3 148Q and parkin WT or V380L, while both proteins showed an overlap in their cytoplasmic distribution (Fig. 2e, f; Supplementary Fig. S9a).

As ataxin-3 was previously characterised as a physical interactor of parkin [12], we analysed potential alterations in its binding to parkin V380L by GFP-Trap-based co-immunoprecipitation using extracts from 293T WT cells, which overexpress both parkin variants together with GFP-tagged ataxin-3 15Q and 77Q (Fig. 2g). While wild-type parkin showed a robust interaction with GFP-Atx3 15Q and 77Q, the V380L variant led to a significant reduction in parkin binding to both ataxin-3 variants by approx. 87% and 62%, respectively (Fig. 2g, h). This impaired interaction may have direct repercussions on the known functional interplay between both proteins, inducing undesired molecular perturbations.

### V380L variant in parkin affects the mitophagy-linked removal of mitochondrial proteins

**Fig. 3:**
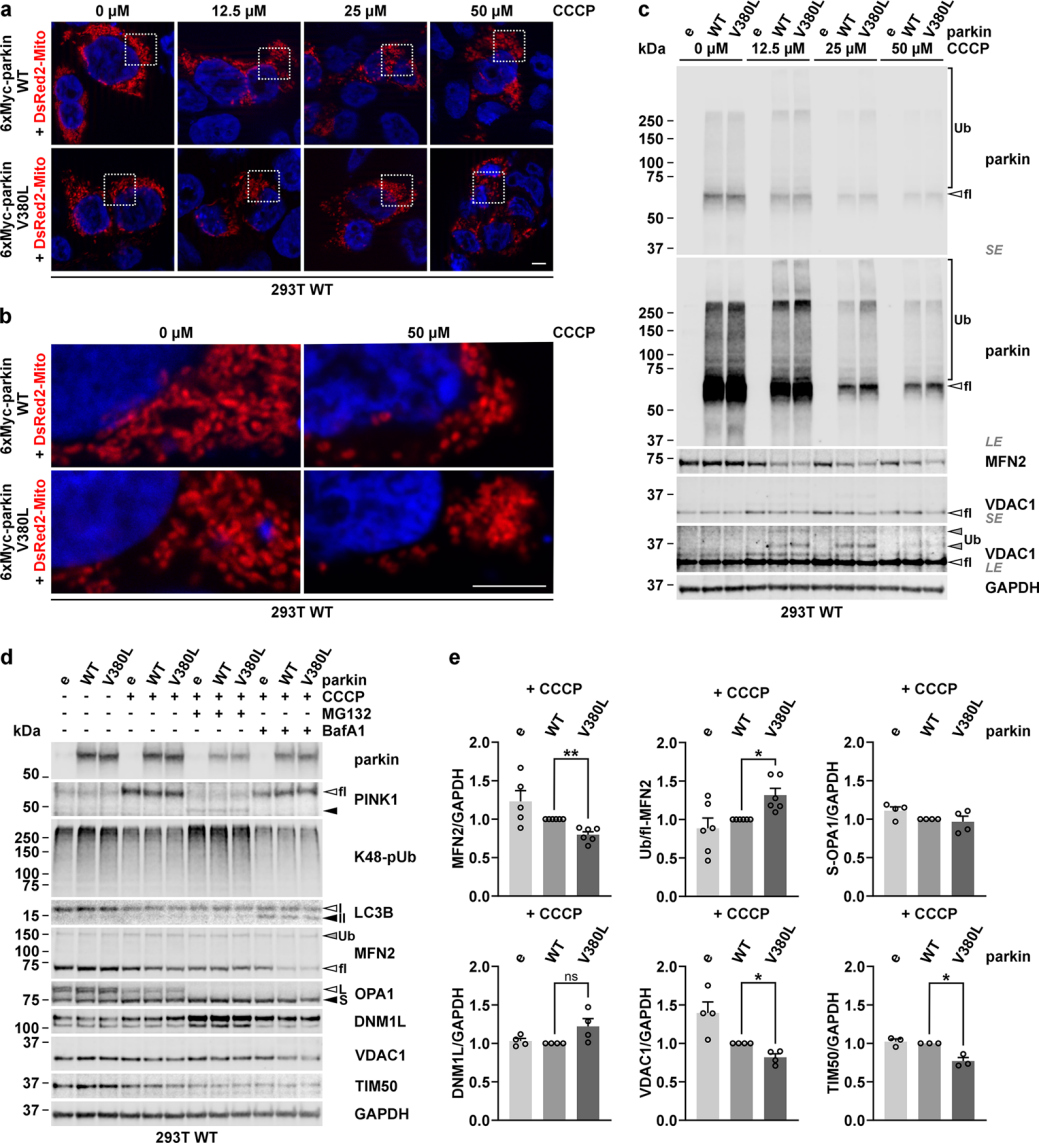
Parkin V380L perturbs mitophagy-linked degradation of mitochondrial proteins. **a** Epi-fluorescence microscopy of 293T WT cells, transfected with 6xMyc-parkin WT or V380L and treated with different concentrations of CCCP for 24 h. Mitochondria were visualised by co-expressing DsRed2-Mito (red). Cells overexpressing parkin WT or V380L show a CCCP concentration-dependent fragmentation and loss of mitochondria. DAPI was used as a nuclear counterstain (blue). Magnifications of areas marked with white dashed boxes can be found in Supplementary Fig. S8a. Scale bar = 5 µm. **b** Confocal microscopy of 293T WT cells co-expressing DsRed2-Mito (red) and 6xMyc-parkin WT or V380L, and treated with 50 µM CCCP for 24 h. DAPI was used as a nuclear counterstain (blue). Scale bar = 5 µm. **c** Western blotting of 293T WT cells expressing 6xMyc-parkin WT or V380L, or transfected with an empty vector (e), and treated with various CCCP concentrations 24 h prior to harvest. Membranes were probed with antibodies against parkin, mitofusin-2 (MFN2), and voltage-dependent anion channel 1 (VDAC1), and demonstrate that mitophagy induction using CCCP shows a concentration-dependent ubiquitination and lowering of overexpressed parkin and other mitochondrial marker proteins. GAPDH served as loading control. White arrowheads indicate full-length, unmodified protein forms. Grey arrowheads show ubiquitinated (Ub) forms of VDAC1. Brackets mark Ub-parkin. *SE*, short exposure, *LE*, long exposure. **d** Western blotting of 293T WT cells expressing 6xMyc-parkin WT or V380L, or transfected with an empty vector (e), and treated with 12.5 µM CCCP 24 h prior to harvest. Cells were additionally incubated with proteasome inhibitor MG132 or autophagy inhibitor bafilomycin A1 (BafA1). Membranes were probed with antibodies against parkin, PINK1, mitofusin-2 (MFN2), optic atrophy protein 1 (OPA1), dynamin 1-like protein (DNM1L), voltage-dependent anion channel 1 (VDAC1), or translocase of inner mitochondrial membrane 50 (TIM50), demonstrating that parkin V380L aggravates loss of certain mitochondrial marker proteins upon CCCP-induced mitophagy. Successful treatment with MG132 or BafA1 was monitored by detecting K48-polyubiquitin chains (K48-pUb) and LC3B-II. GAPDH served as loading control. White arrowheads indicate full-length (fl)/long (L) forms of PINK1, MFN2 or OPA1 as well as LC3B-I (I). Grey arrowhead points to ubiquitinated (Ub) MFN2. Black arrowheads show cleaved/short (S) forms of PINK1 or OPA1 as well as LC3B-II (II). **e** Densitometric quantification of protein levels upon CCCP treatment, normalised to GAPDH. Ub-MFN2 was normalised to fl-MFN2. Within each experimental replicate, values were additionally normalized to the parkin WT/+CCCP control. *n* = 3–6. Bars represent mean + s.e.m. One sample *t*-test; *P ≤ 0.05; **P ≤ 0.01; *ns* not significant

**Fig. 4:**
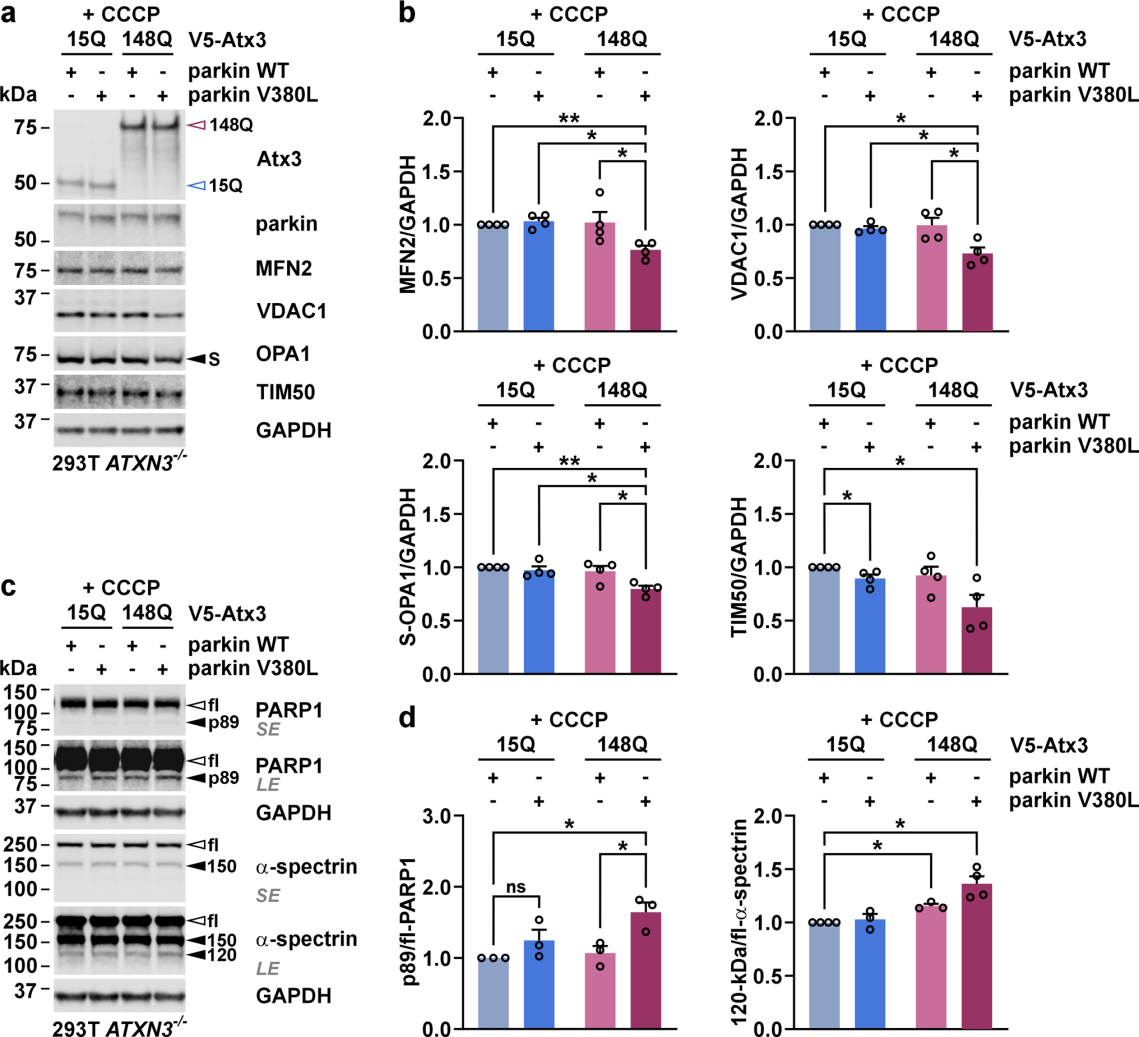
Parkin V380L-induced disturbances in mitophagy persist upon expression of polyQ-expanded ataxin-3, leading to increased cell death markers. **a** Western blotting of 293T *ATXN3*^−/−^ cells co-expressing V5-Atx3 15Q or 148Q and 6xMyc-parkin WT or V380L, treated with 12.5 µM CCCP 24 h prior harvest. Membranes were probed with antibodies against ataxin-3, parkin, MFN2, VDAC1, OPA1, and TIM50. Upon mitophagy induction, increased parkin V380L-linked loss of mitochondrial markers is alleviated by co-expression of wild-type ataxin-3, while the effects persist in presence of the polyQ-expanded protein. GAPDH served as loading control. Blue-rimmed arrowhead indicates ataxin-3 15Q and purple-rimmed arrowhead shows ataxin-3 148Q. Black arrowhead shows short (S) form of OPA1. **b** Densitometric quantification of protein levels upon CCCP treatment, normalised to GAPDH. Within each experimental replicate, values were additionally normalised to the control Atx3 15Q/parkin WT/+CCCP. *n* = 4. Bars represent mean + s.e.m. One sample *t*-test (comparisons to the control group) or one-way ANOVA (comparisons between the other groups); *P ≤ 0.05; **P ≤ 0.01. **c** Western blot analysis of poly [ADP-ribose] polymerase 1 (PARP1) and ɑ-spectrin cleavage as cell death markers. White arrowheads indicate full-length (fl) PARP1 or fl-ɑ-spectrin. Black arrowheads show the apoptosis-associated p89 cleavage product of PARP1 and ɑ-spectrin breakdown products at 150 kDa (150) and 120 kDa (120). Elevated levels of caspase cleavage-derived PARP1 p89 and 120-kDa-ɑ-spectrin occur in parkin V380L and polyQ-expanded but not wild-type ataxin-3 co-expressing cells upon mitophagy induction. GAPDH served as loading control. *SE*, short exposure, *LE*, long exposure. **d** Densitometric quantification of apoptosis-associated PARP1 p89 and 120-kDa-ɑ-spectrin levels upon CCCP treatment, both normalised to the respective full-length protein. Within each experimental replicate, values were additionally normalised to the control Atx3 15Q/parkin WT/+CCCP. *n* = 3–4. Bars represent mean + s.e.m. One sample *t*-test (comparisons to the control group) or one-way ANOVA (comparisons between the other groups); *P ≤ 0.05

**Fig. 5:**
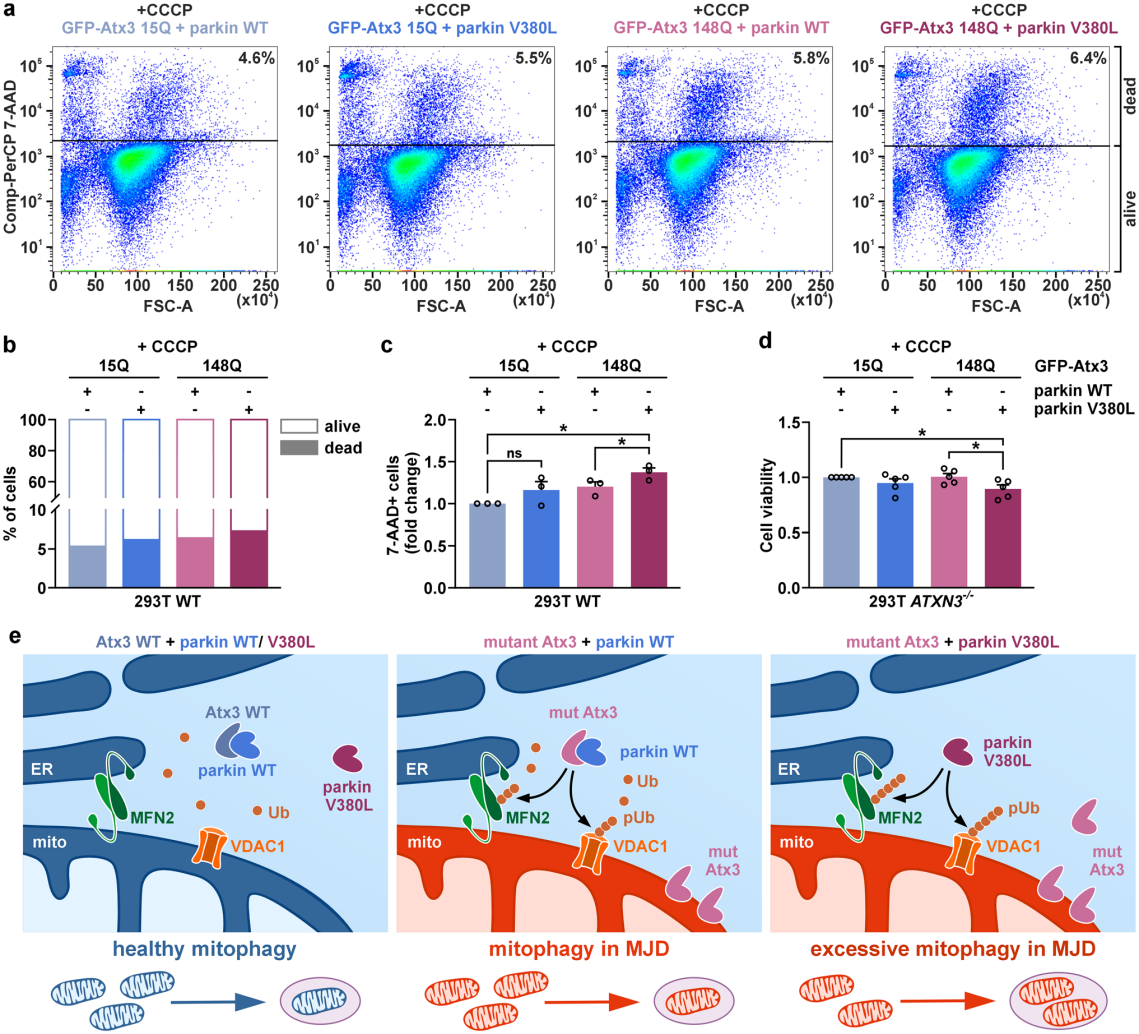
Parkin V380L further compromises viability of cells expressing polyQ-expanded ataxin-3 upon induction of mitophagy. **a** FACS-based cell death analysis of 293T WT cells co-expressing V5-Atx3 15Q or 148Q and 6xMyc-parkin WT or V380L, treated with 12.5 µM CCCP for 24 h prior assessment. Dead cells were stained using 7-aminoactinomycin D (7-AAD). Representative density plots and percentages for the population of dead cells for one experimental replicate are shown. Spectrally compensated signals for 7-AAD in the PerCP channel (Comp-PerCP 7-AAD) were plotted against the forward-scatter area (FSC-A). **b** Mean percentages of alive and dead cells are shown. Bars represent mean values of *n* = 3 biological replicates. **c** Fold changes of 7-AAD-positive (7-AAD+) cells were calculated within each experimental replicate by normalising the values to the control Atx3 15Q/parkin WT/+CCCP. Cells co-expressing parkin V380L and polyQ-expanded ataxin-3 show an increase in cell death upon CCCP administration. *n* = 3. Bars show mean + s.e.m. One sample *t*-test (comparisons to the control group) or one-way ANOVA (comparisons between the other groups); *P ≤ 0.05. **d** Resazurin-based viability analysis of 293T *ATXN3*^−/−^ cells co-expressing V5-Atx3 15Q or 148Q and 6xMyc-parkin WT or V380L, treated with 12.5 µM CCCP for 24 h prior assessment. Within each experimental replicate, values were additionally normalised to the control Atx3 15Q/parkin WT/+CCCP. Cells co-expressing parkin V380L and polyQ-expanded ataxin-3 show a reduced cell viability upon CCCP administration. *n* = 4. Bars represent mean + s.e.m. One sample *t*-test (comparisons to the control group) or one-way ANOVA (comparisons between the other groups); *P ≤ 0.05. **e** Schematic summary and suggested pathological model based on observed effects of parkin V380L in MJD. In healthy cells, interactors parkin and ataxin-3 are involved in the basic maintenance of mitochondria, showing a balance between their E3 ubiquitin ligase and deubiquitinase activities. In MJD, mitochondria become compromised by mutant ataxin-3 (mut Atx3), undergoing mitophagy, which is mediated by parkin recruitment, polyubiquitination (pUb) of substrates such as MFN2 and VDAC1, and trimming by ataxin-3. In case of parkin V380L, reduced interaction with ataxin-3 leads to unbalanced polyubiquitination of mitochondrial substrates and thereby excessive mitophagy with negative consequences on neuronal viability. ER, endoplasmic reticulum; mito, mitochondrion

As we detected a reduced interaction between parkin V380L and ataxin-3, we investigated its potential effects on Phosphatase And Tensin Homolog Induced Kinase 1 (PINK1)-parkin-mediated mitophagy [33]. In a first approach, we transfected 293T cells with parkin WT or V380L and depolarized mitochondria by administering different concentrations of the protonophore CCCP to induce mitophagy. Using epi- and confocal microscopy as well as western blotting, we analysed effects on mitochondrial morphology and marker proteins. Cells co-expressing DsRed2-Mito for visualising mitochondria showed the earlier described induction of mitochondrial fragmentation, formation of spheroids and widespread depletion [9, 31], most prominent at 50 µM CCCP upon expression of both parkin variants (Fig. 3a, b, Supplementary Fig. S10a, b). Several mitochondria-associated markers (see overview in Supplementary Fig. S11a) are known to be affected in the process of mitophagy, including PINK1, which accumulates at mitochondrial membranes in its uncleaved, full-length (fl-PINK1) form to recruit parkin [32], and mitofusin-1 (MFN1), -2 (MFN2), or voltage-dependent anion channel 1 (VDAC1), which are ubiquitinated by parkin for proteasomal or autophagosomal breakdown [14, 46]. Western blotting analysis showed a CCCP treatment-induced shift from full-length (fl-)parkin WT or V380L to its modified, ubiquitinated forms, an effect described previously [14, 27], already detectable at 12.5 µM CCCP, and its depletion at higher concentrations (Fig. 3c, Supplementary Fig. S11b). Moreover, we observed the accumulation of fl-PINK1, as well as a drop of translocase of inner mitochondrial membrane 50 (TIM50) levels, and an Optic atrophy 1 (OPA1) transition from its long (L-) to its proteolyzed short (S-) form, independently of the parkin overexpression, while cytochrome c (Cyt c), translocase of the outer mitochondrial membrane 20 (TOM20), and citrate synthase (CS) levels remained unchanged (Supplementary Fig. S11c). On the other hand, an obvious lowering of MFN1, MFN2 and VDAC1 did only take place when either parkin WT or V380L were overexpressed, while dynamin 1-like protein (DNM1L) levels rose (Fig. 3c, Supplementary Fig. S11c). To confirm whether the observed effects depend on proteasomal or autophagosomal degradation as previously reported [14, 28, 46], we repeated our experiments by including co-treatments with MG132 or epoxomicin to inhibit proteasomes, or bafilomycin A1 (BafA1) for inhibiting autophagy (Fig. 3d; Supplementary Fig. S11d-f). Western blotting showed that degradation of MFN2 upon presence of parkin was partly rescued by proteasomal inhibition using MG132 or epoxomicin, while BafA1 failed to do so. L- to S-OPA1 transition remained unaffected, as it occurs independent of both degradative pathways [3]. Also, neither MG132 nor BafA1 treatments prevented loss of TIM50. Noteworthily, administration of MG132 counteracted the accumulation of uncleaved PINK1 but led to an increase of DNM1L, latter being a known substrate of proteasomal degradation [51]. Importantly, further analysis indicated differences between parkin WT and V380L in the turnover of its specific substrates and other mitochondrial proteins. While no statistically significant changes in S-OPA1 or DNM1L levels occurred, quantitative analysis revealed an increased lowering of MFN2 in the presence of parkin V380L, accompanied by higher levels of its presumably ubiquitinated form (Ub-MFN2), as well as a stronger drop of VDAC1 and TIM50 levels (Fig. 3e). To investigate these effects in an MJD-relevant context, we replicated our experiments upon co-expression of V5-tagged ataxin-3 15Q or 148Q in 293T *ATXN3*^−/−^ cells. The previously observed CCCP-induced lowering of MFN2 and VDAC1 levels for parkin V380L were recapitulated upon ataxin-3 148Q but not ataxin-3 15Q co-expression, confirming the relevance of the parkin variant for MJD (Fig. 4a, b). The same effects were also observed for TIM50 and S-OPA1 (Fig. 4a, b), while DNM1L levels did not fluctuate between experimental groups upon CCCP administration (Supplementary Fig. S12a, b). To evaluate whether the observed increase in mitochondrial protein loss had repercussions on cell viability, we analysed caspase-dependent poly [ADP-ribose] polymerase 1 (PARP1) and ɑ-spectrin cleavage as markers of apoptosis. Both markers indicated an increased cell death, as levels of the PARP1 p89 breakdown product and cleaved 120-kDa ɑ-spectrin were elevated (Fig. 4c, d). Using a FACS-based analysis, performed by staining dead cells with 7-AAD under the same treatment conditions, we confirmed an increased cell death in GFP-Atx3 148Q and parkin V380L co-transfected 293T WT cells upon administration of CCCP (Fig. 5a–c). In line with this, a resazurin-based assay showed a decreased cell viability of 293T *ATXN*3^−/−^ cells co-expressing GFP-Atx3 148Q and parkin V380L after a 24-h incubation with CCCP (Fig. 5d).

Taken together, we identified that parkin V380L aggravated the mitophagy-associated depletion of mitochondrial proteins in the presence of polyQ-expanded ataxin-3, indicating detrimental consequences on the molecular and cellular pathology in MJD patients and explaining the observed earlier AAO in homozygous carriers of this parkin variant.

## Discussion

By analysing a large cohort of MJD patients, we identified the SNP rs1801582 in *PRKN* as a modifier of the disease AAO. This genetic variant causes a V380L amino acid exchange in the encoded E3 ubiquitin ligase parkin, a known interaction partner of the DUB ataxin-3. While our functional investigations neither detected reciprocal effects on the levels of both proteins nor alterations in the aggregation of polyQ-expanded ataxin-3, it did reveal a significant impact on the interaction of both proteins and repercussions on the performance of parkin-linked mitophagy, leading to an excessive removal of mitochondrial proteins and thus mitochondria (summarised in Fig. 5e), with consequences on cell viability.

Aside from the known negative correlation between the CAG repeat length and the AAO in MJD [17], genetic modifiers likely account for the high variability in disease onset between individuals with similar repeat numbers as observed in various polyQ diseases [7, 23]. With a frequency of 1/1000 base pairs, SNPs represent the most common genetic variation in the human genome [8, 44], and evidentially contribute to the AAO in MJD [2, 5, 37].

To widen the known spectrum of genetic modifiers of MJD, we examined the three most frequent missense variants in the *PRKN* gene, which encodes the well-described ataxin-3 interaction partner parkin [12]. After a pilot analysis, we excluded two rarer variants and then genotyped more than 900 MJD patients grouped into three major sub-cohorts for SNP rs1801582 in exon 10. Analysing differences in the AAO across rs1801582 genotypes by direct comparison as well as using an interactive model of multivariate linear regression to account for an interrelationship between CAG repeat numbers in *ATXN3* and the SNP rs1801582 genotype, we discovered that patients homozygous for the minor allele showed an earlier AAO by about 3 years when combining all three sub-cohorts. This difference corresponds to observations made in comparable studies for other genetics modifiers [25, 37].

While our subsequent functional analysis of the SNP in an MJD cell model did not demonstrate any repercussions on soluble ataxin-3 levels or aggregated forms of the polyQ-expanded protein upon parkin V380L co-expression, we could detect a reduced binding of ataxin-3 to the parkin variant. The V380L exchange lies at the border between the IBR and the repressor domain of parkin (REP), and thus between its IBR and RING2 domains. The variant was suggested to induce conformational changes [4], which likely explain the observed impairment of the parkin:ataxin-3 interaction in our co-immunoprecipitation experiments. It may also be a basis for repercussions on ubiquitination abilities of parkin, which was implied in a study on thyroid Hürthle cell tumours, where the parkin V380L variant resulted in an impaired mitophagic turnover [20]. In contrast to this, our experiments showed an exacerbated CCCP-induced lowering of different mitochondrial markers, including the well-described parkin substrates MFN2 and VDAC1 in the context of mitophagy [14, 28, 46], in parkin V380L-expressing cells. This decrease was maintained when the variant was co-expressed with polyQ-expanded but not wild-type ataxin-3. Noteworthily, a previous study showed that VDAC1 is deubiquitinated by ataxin-3 [16]. Consequently, a variant-triggered impairment of the interplay between the E3 ubiquitin ligase parkin and the DUB ataxin-3 may indicate a pathologically increased mitophagy under stress conditions.

While an impairment of a proper mitochondrial removal by autophagic mechanism is seen as a burden in the molecular pathogenesis of Parkinson’s disease and other diseases [13, 33, 57], excessive mitophagy has been suggested as a driver of neurodegeneration in Huntington’s disease [15]. In general, destabilising the balanced mechanism of mitophagy in either direction is known to result in cellular stress and degeneration [10]. This basic concept may explain the increased cell death markers and decreased viability observed in our cell model, thus offering a functional explanation for the observed earlier AAO of MJD patients homozygous for the minor allele of SNP rs1801582 in *PRKN*.

Our findings show statistical significance despite the low abundance of homozygous carriers and the low prevalence of the disease. While same trends for a negative impact of the rs1801582 C/C genotype on the AAO were observed throughout our analyses, certain limitations of our study persist, as only a combination of three investigated sub-cohorts yielded statistically significant results. The lack of detailed pedigree structure information for our cohorts led to the application of a family factor to compensate for a potential bias of relatedness between MJD patients included in this study, which might represent a suboptimal approach for correcting the data. Finally, as the analysed MJD patient samples are mainly of European origin, correlation to subjects with different geographic origins should be approached with caution. Therefore, our study awaits external replication in a different cohort and a potential translation to other polyglutamine diseases.

However, the broader relevance of our dataset, which is strongly supported by our functional data, is evident for our understanding of the molecular pathogenesis in MJD, as well as for affected patients. With this in mind, our study highlights the importance of performing comparative and transnational studies on genetic modifiers in rare diseases, which may reveal pathomechanistic links, as shown for MJD and Parkinson’s disease in our work.

Taken together, we identified the parkin V380L variant as a novel genetic modifier with a deciphered, mitophagy-related contribution to the molecular pathogenesis in MJD, which may allow for a more precise prognostic evaluation of patients suffering from this yet incurable disorder, and reveal a potential target for the development of a treatment strategy.

### Supplementary Information

Below is the link to the electronic supplementary material.

Supplementary file1 (PDF 1669 kb)

## Data Availability

The datasets generated or analysed during this study are included in this published article and its supplementary files. Details on Fiji-based macro for morphological analysis of protein aggregates will be shared upon request.
