# Supplementary material for: The parkin V380L variant is a genetic modifier of Machado–Joseph disease with impact on mitophagy
Source: Acta Neuropathol. 2024 Aug 1;148(1):14. doi: 10.1007/s00401-024-02762-6 (PMC11294389; doi:10.1007/s00401-024-02762-6)
Supplement: Supplementary file 1 — Supplementary file1 (PDF 1669 kb) [file 401_2024_2762_MOESM1_ESM.pdf]

Supplementary file 1

The parkin V380L variant is a genetic modifier of Machado-Joseph disease with impact on mitophagy

Jonasz J. Weber[1,2,*], Leah Czisch[1,*], Priscila Pereira Sena[1], Florian Fath[1,2], Chrisovalantou Huridou[1,2], Natasa Schwarz[1], Rana D. Incebacak Eltemur[1,2], Anna Würth[1], Daniel Weishäupl[1], Miriam Döcker[1], Gunnar Blumenstock[3], Sandra Martins[4,5], Jorge Sequeiros[4,6], Guy A. Rouleau[7], Laura Bannach Jardim[8,9], Maria-Luiza Saraiva-Pereira[9,10], Marcondes C. França Jr.[11], Carlos R. Gordon[12], Roy Zaltzman[12], Mario R. Cornejo-Olivas[13], Bart P. C. van de Warrenburg[14], Alexandra Durr[15,16], Alexis Brice[15,16], Peter Bauer[1,17,18], Thomas Klockgether[19,20], Ludger Schöls[21,22], Olaf Riess[1], The EUROSCA Network[23], Thorsten Schmidt[1]

[*]These authors contributed equally to this work.

Affiliations:

[1] Institute of Medical Genetics and Applied Genomics, University of Tübingen, 72076 Tübingen, Germany
[2] Department of Human Genetics, Ruhr University Bochum, 44801 Bochum, Germany
[3] Department of Clinical Epidemiology and Applied Biometry, University of Tübingen, 72076 Tübingen, Germany
[4] i3S - Instituto de Investigação e Inovação em Saúde, Universidade do Porto, 4200-135 Porto, Portugal
[5] IPATIMUP - Institute of Molecular Pathology and Immunology, University of Porto, 4200-135 Porto, Portugal
[6] ICBAS School of Medicine and Biomedical Sciences, University of Porto, 4050-313 Porto, Portugal
[7] Department of Neurology and Neurosurgery & The Neuro (Montreal Neurological Institute-Hospital), McGill University, H3A 1A1 Montréal, Canada
[8] Departamento de Medicina Interna, Faculdade de Medicina, Universidade Federal do Rio Grande do Sul, 90035-903, Porto Alegre, Brazil
[9] Serviço de Genética Médica, Hospital de Clínicas de Porto Alegre, 90035-903, Porto Alegre, Brazil
[10] Departamento de Bioquímica, Universidade Federal do Rio Grande do Sul, 90035-003Porto Alegre, Brazil
[11] Universidade Estadual de Campinas (UNICAMP), 13083-970 Campinas, Brazil
[12] Department of Neurology, Tel Aviv University, 69978 Tel Aviv, Israel
[13] Neurogenetics Research Center, Instituto Nacional de Ciencias Neurológicas, 15003 Lima, Peru
[14] Department of Neurology, Donders Institute for Brain, Cognition, and Behaviour, Radboud University Medical Center, 6525 Nijmegen, The Netherlands
[15] 4 AP-HP, Groupe Hospitalier Pitié-Salpêtrière, Department of Genetics and Cytogenetics, F-75013, Paris, France
[16] Sorbonne Université, Institut du Cerveau - Paris Brain Institute - ICM, Inserm, CNRS, APHP, University Hospital Pitié-Salpêtrière, 75013, Paris, France
[17] Centogene GmbH, 18055 Rostock, Germany
[18] Clinic for Internal Medicine, Department of Hematology, Oncology, Palliative Medicine, University Medicine Rostock, 18057 Rostock, Germany
[19] German Center for Neurodegenerative Diseases (DZNE), 53127 Bonn, Germany
[20] Department of Neurology, University Hospital Bonn, 53127 Bonn, Germany
[21] Department of Neurology and Hertie-Institute for Clinical Brain Research, University of Tübingen, 72076 Tübingen, Germany
[22] German Center of Neurodegenerative Diseases (DZNE), 72076 Tübingen, Germany
[23] See Supplementary data for details of The EUROSCA Network.

Correspondence should be addressed to:
Dr. Thorsten Schmidt, PhD, MME, Institute of Medical Genetics and Applied Genomics, University of Tübingen, Nägelestraße 5, 72074 Tübingen, Germany; Phone: +49 7071 29 72277; Fax: +49 7071 29 5228; e-mail: thorsten.schmidt@med.uni-tuebingen.de

Supplementary figures and figure legends

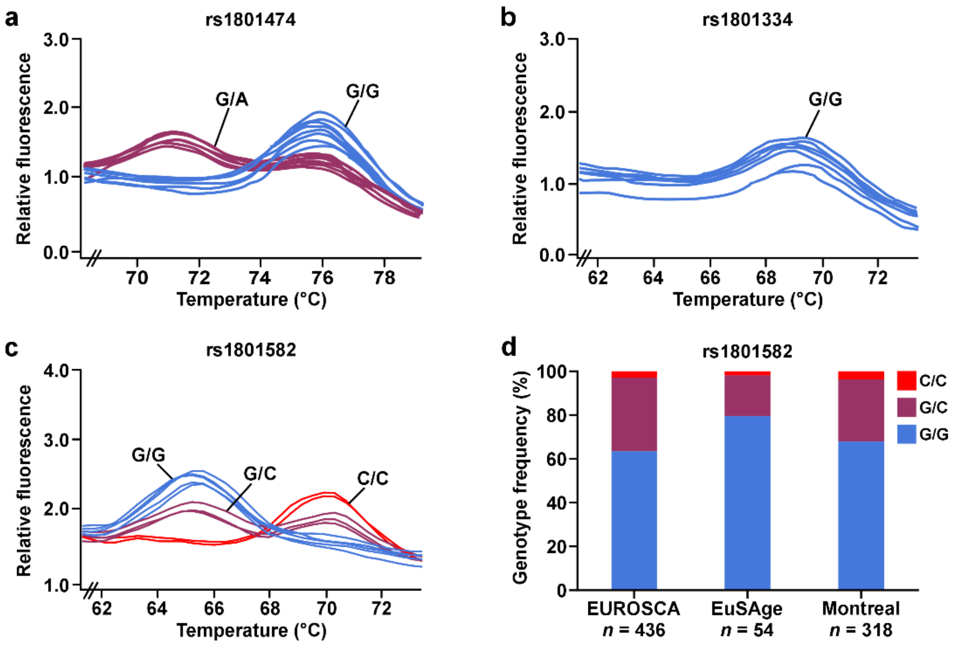

Supplementary Fig. S1 High-resolution melting-based genotyping of *PRKN* SNPs in MJD patients. High-resolution melting analysis employing unlabelled probes for detecting *PRKN* SNP genotype-specific melting peaks. (**a**) Melting curve analysis for rs1801474 (c.601G>A, p.S167N) in *PRKN* exon 4. MJD patient samples with homozygous genotype G/G formed a single peak at 76°C melting temperature. Heterozygous samples with the genotype G/A showed a double-peak, at approximately 71°C and 76°C melting temperature, respectively. (**b**) Melting curve analysis for rs1801334 (c.1281G>A, p.D394N) in *PRKN* exon 11. MJD patient samples carrying the homozygous genotype G/G formed a peak at about 69°C. No samples carrying genotype A/A were found in the examined cohort using high-resolution melting. (**c**) Melting curve analysis for rs1801582 (c.1239G>C, p.V380L) in *PRKN* exon 10. Samples with genotype G/G showed a melting peak at about 65 °C and with genotype C/C a melting peak at about 70 °C. Samples with the heterozygous genotype G/C showed both melting peaks at 65 °C and 70 °C. (**d**) Genotype frequencies of rs1801582 in *PRKN* exon 10 divided in the analysed EUROSCA, EuSAge, and Montreal sub-cohorts. No significant differences in the geographical distribution of the genotypes were detected ($P = 0.109$; Fisher-Freeman-Halton exact test)

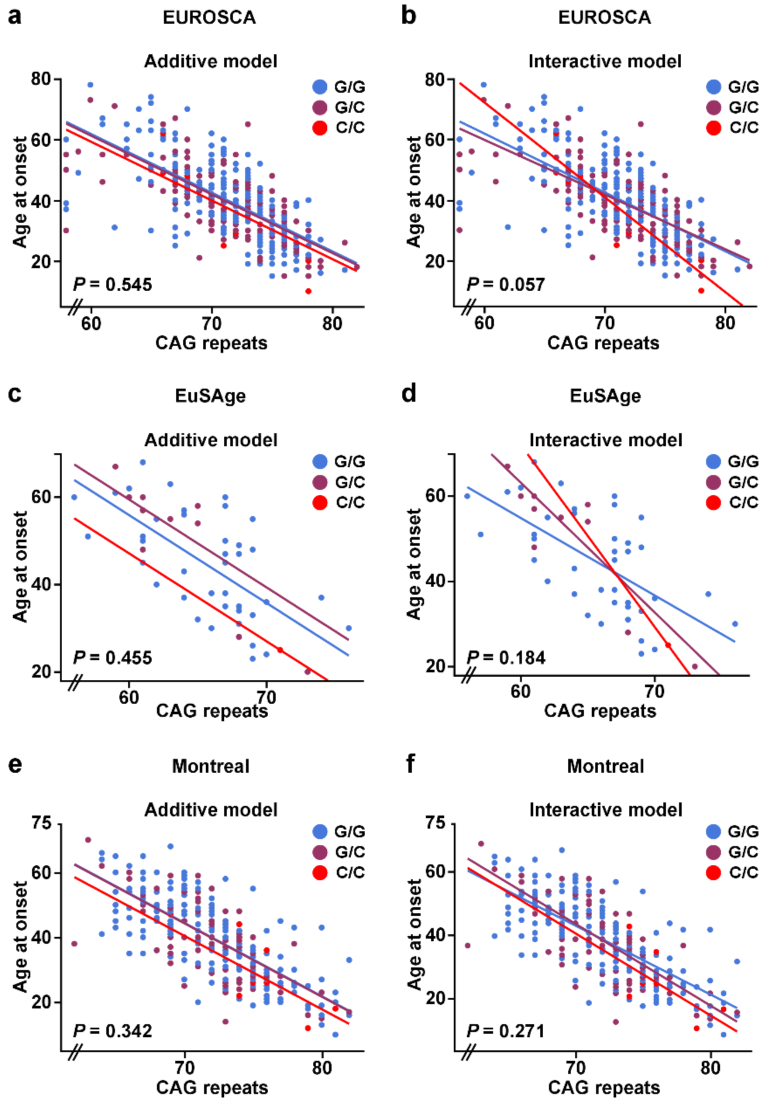

Supplementary Fig. S2 Additive and interactive models of multivariate linear regression analysis of EUROSCA, EuSAge, and Montreal sub-cohorts. (**a**, **c**, **e**) Additive model of multivariate linear regression for the EUROSCA, EuSAge, and Montreal sub-cohorts for analysing the impact of the *PRKN* rs1801582 genotype. Linear regression of the C/C genotype indicates an earlier age at onset through all cohorts, while not reaching statistical significance. (**b**, **d**, **f**) Interactive model of multivariate linear for the EUROSCA, EuSAge, and Montreal sub-cohorts for analysing the impact of the *PRKN* rs1801582 genotype. Combination of higher CAG repeat numbers with the C/C genotype showed trends towards an earlier AAO, most prominent for the EUROSCA and EuSAge sub-cohorts.

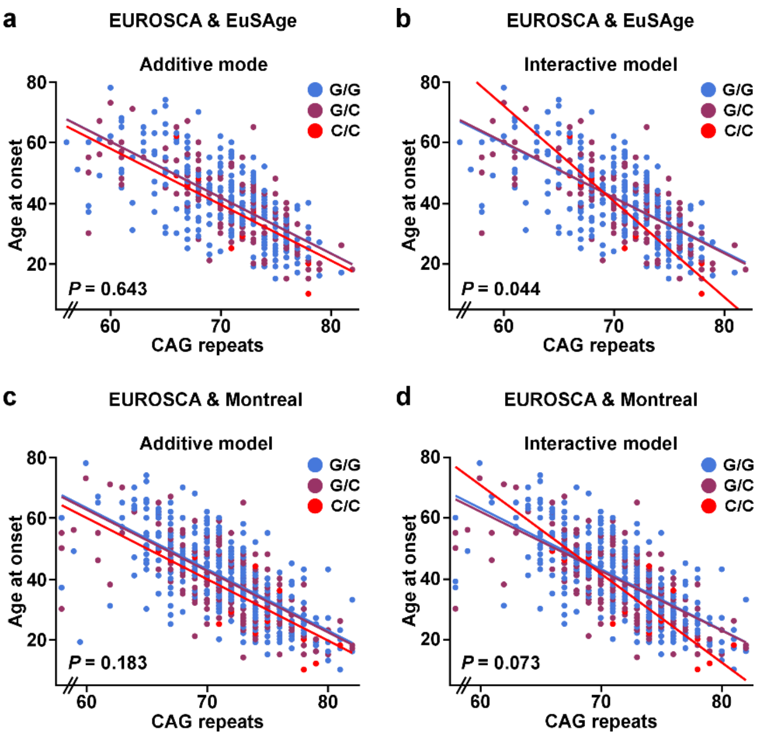

Supplementary Fig. S3 Additive and interactive models of multivariate linear regression analysis for combinations of two MJD sub-cohorts. (**a**, **b**) Additive and interactive model of multivariate linear regression for a combination of the EUROSCA and EuSAge sub-cohorts for analysing the impact of the *PRKN* rs1801582 genotype. The interactive model shows a significant effect of the C/C genotype regression on the age at onset ($P = 0.044$). (**c**, **d**) Additive and interactive model of multivariate linear regression for a combination of the EUROSCA and Montreal sub-cohorts for analysing the impact of the *PRKN* rs1801582 genotype. The interactive model shows a strong trend towards an effect of the C/C genotype on the age at onset ($P = 0.076$)

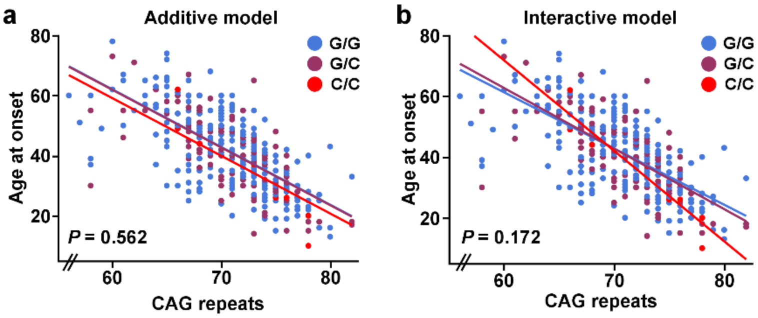

Supplementary Fig. S4 Sensitivity analysis for additive and interactive models of multivariate linear regression. (**a**, **b**) A sensitivity analysis was performed for the additive and interactive model of multivariate linear regression for the combined MJD cohort by exchanging the family factor against a random selection of one individual per MJD family. While comparable trends of the *PRKN* rs1801582 C/C genotype remained, the effects did not reach statistical significance, with the interaction between the polymorphism and the CAG repeat length reaching a *P-value of* 0.172

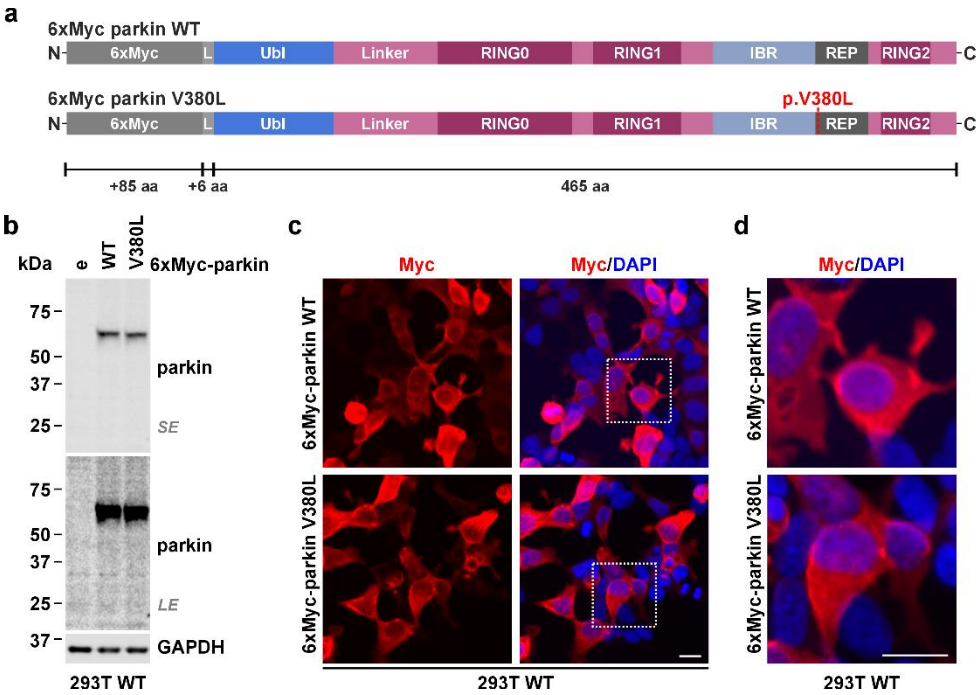

Supplementary Fig. S5 Overexpression of parkin WT and V380L in 293T cells. (**a**) Schematic representation of N-terminally 6xMyc-tagged parkin wild-type (WT) or parkin featuring a valine to leucine exchange at amino acid position 380 (V380L), located between the in-between-ring domain (IBR) and the repressor element of parkin (REP). Ubl, ubiquitin-like domain; RING, really-interesting-new-gene domain. L, linker sequence between parkin and its 6xMyc tag. (**b**) Western blotting of 293T WT cells, transfected with an empty vector (e) or overexpressing 6xMyc-parkin WT or V380L, demonstrated that the overexpression of parkin variants leads to robust protein levels of parkin while endogenous parkin is undetectable. GAPDH served as loading control. *SE*, short exposure; *LE*, long exposure. (**c**) Fluorescence microscopy of 293T WT cells, transfected with 6xMyc-parkin WT or V380L. Parkin expression (red) was visualised using a Myc tag-specific antibody, revealing a similar cytoplasmic localization of the parkin variants. DAPI was used as a nuclear counterstain (blue). (**d**) Magnifications of areas marked with white dashed boxes in **c**. Scale bars = 20 μm

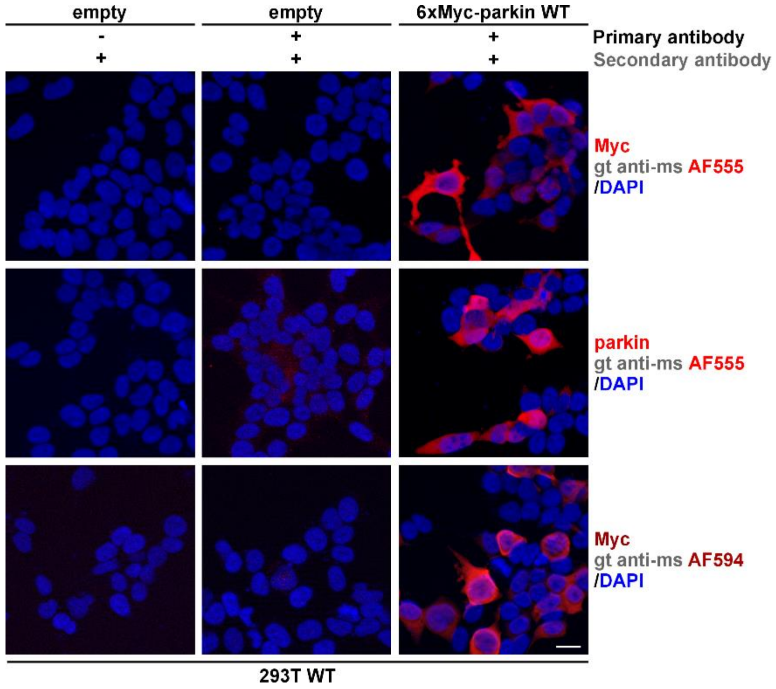

Supplementary Fig. S6 Control immunostainings of 293T cells. Fluorescence microscopy of 293T WT cells, transfected with empty vector (empty) or 6xMyc-parkin WT. Immunostaining was performed using Myc tag- and parkin-specific primary antibodies and the respective Alexa Fluor 555 (AF555) or 594 (AF594)-labelled secondary antibodies. Specificity of primary and secondary antibodies was confirmed by staining cells only with the secondary antibody (first column) or cells without overexpression of parkin (second column). Comparison between the negative controls in the first and second columns confirms the specificity of the immunostaining procedure for overexpressed Myc-tagged parkin as shown in the third column (red signals). DAPI was used as a nuclear counterstain (blue). Scale bar = 20 µm

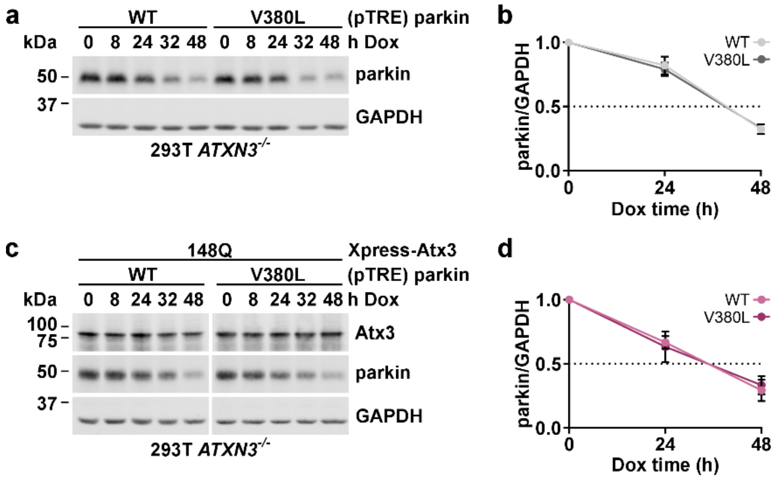

Supplementary Fig. S7 Parkin V380L shows the same stability as the wild-type form. Protein stability was assessed using a cell-based Tet-off system. (**a**) Western blot analysis of 293T *ATXN3*$^{-/-}$ cells transfected with pTRE-parkin WT or V380L responder constructs and a pTET-RCA2 vector for 72 h. Expression of parkin was terminated by the administration of doxycycline (Dox) at desired time points. Membranes were probed with a parkin antibody. Both parkin variants show a similar time-dependent drop in their levels, indicating comparable protein stability. GAPDH served as loading control. (**b**) Densitometric quantification of parkin normalised to GAPDH. Within each experimental replicate, values were additionally normalized to the time point 0 h. n = 3. Data points represent mean ± s.e.m. (**c**) Western blot analysis of 293T *ATXN3*$^{-/-}$ transfected with pTRE-parkin WT or V380L responder constructs and a pTET-RCA2 vector, under overexpression of Xpress-Atx3 148Q for 72 h. Expression of parkin was terminated by the administration of Dox at desired time points. Membranes were probed with antibodies against ataxin-3 or parkin. Both parkin variants do not show differences in their protein stability in presence of polyQ-expanded ataxin-3. GAPDH served as loading control. (**d**) Densitometric quantification of parkin normalised to GAPDH. Within each experimental replicate, values were additionally normalized to the time point 0 h. n = 3. Data points represent mean ± s.e.m.

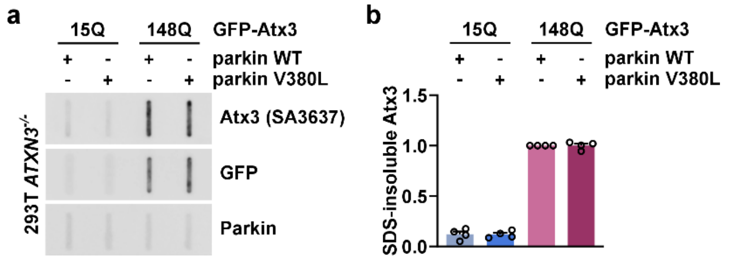

Supplementary Fig. S8 Parkin V380L does not influence aggregation of polyQ-expanded GFP-Atx3. (**a**) Filter retardation analysis of 293T *ATXN3*$^{-/-}$ cells expressing parkin WT or V380L in combination with wild-type (15Q) or polyQ-expanded (148Q) GFP-tagged ataxin-3. SDS-insoluble protein species were detected using the C-terminally binding ataxin-3-specific antibody SA3637 and a GFP-tag specific antibody, demonstrating that aggregate levels of GFP-tagged polyQ-expanded ataxin-3 remain unaltered upon overexpression of parkin variants. Membranes were additionally stained with an antibody against parkin. (**b**) Densitometric quantification of SDS-insoluble ataxin-3 detected with antibody SA3637. Within each experimental replicate, values were additionally normalized to the Atx3 148Q/parkin WT control. *n* = 4. Bars represent mean + s.e.m.

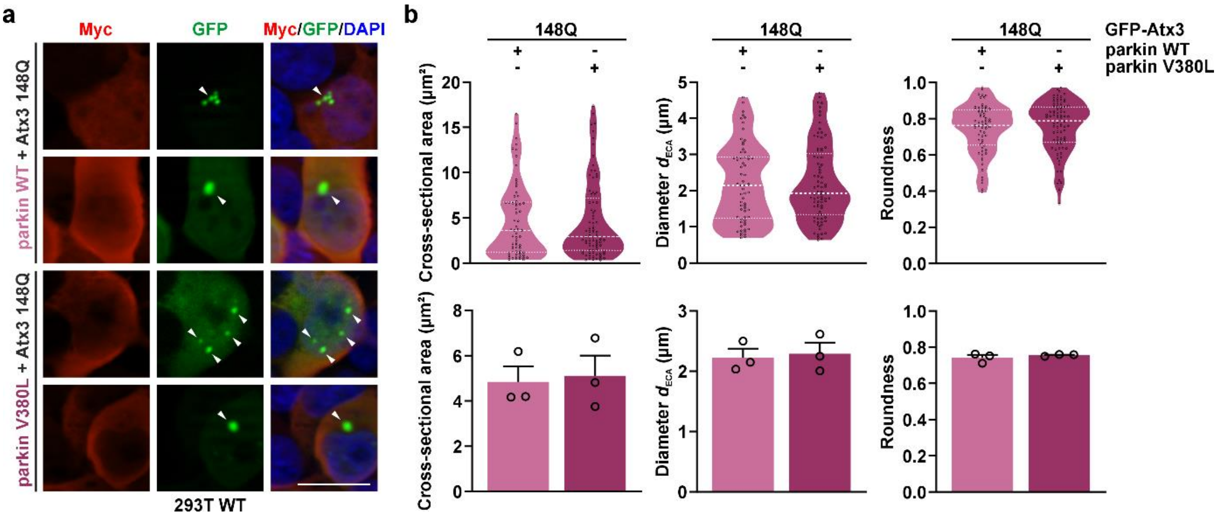

Supplementary Fig. S9 Parkin V380L does not alter morphological aggregate features of polyQ-expanded GFP-Atx3. (**a**) Fluorescence microscopy of 293T WT cells, co-transfected with 6xMyc-parkin WT or V380L and EGFP-Atx3 148Q for analysing morphological characteristics of ataxin-3 aggregates. Parkin was detected using a Myc tag-specific antibody (red). Ataxin-3 and ataxin-3-specific aggregates were visualised via the GFP tag (green). DAPI was used as a nuclear counterstain (blue). White arrowheads indicate aggregates formed by GFP-Atx3 148Q. Scale bar = 20 µM. Representative images in rows 1 and 3 show rather smaller aggregates, while pictures in row 2 and 4 feature larger aggregates. (**b**) Fiji-based quantitative analysis was performed to evaluate effects of the parkin V380L variant on cross-sectional area, equivalent circular area diameter (d_{ECA}) and roundness of ataxin-3 aggregates captured by fluorescence microscopy. Top row shows truncated violin plots for every single aggregate analysed (n_{WT} = 65 and n_{V380L} = 81 aggregates), with dashed white lines indicating the median, and dotted white lines the 25% and 75% percentile, respectively. Bottom row features the means of n = 3 biological replicates. Bars represent mean + s.e.m. Parkin V380L does not lead to any statistically significant alterations of the analysed morphological features of ataxin-3 aggregates compared to the wild-type counterpart

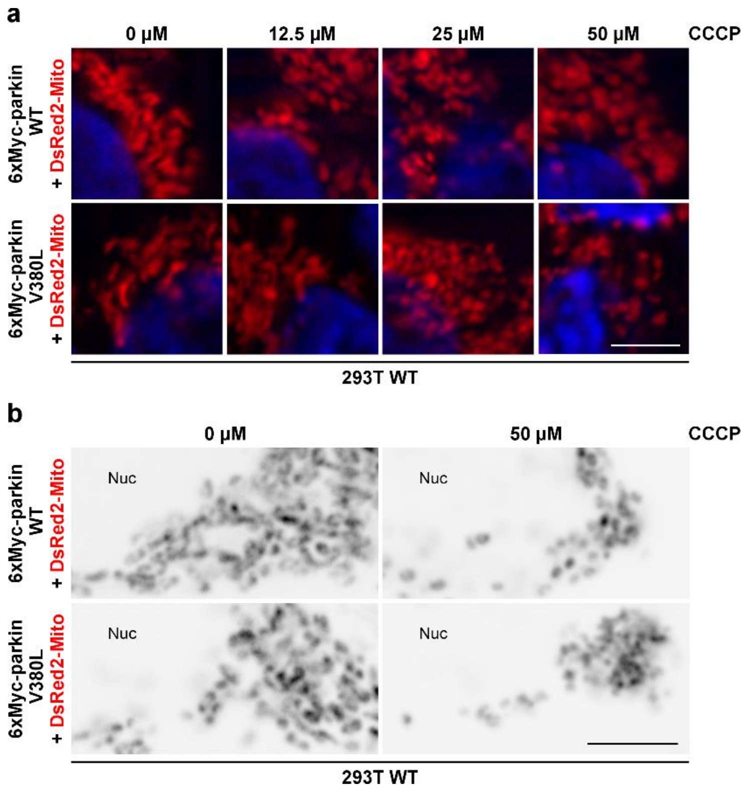

Supplementary Fig. S10 Fluorescence microscopy demonstrates mitochondrial fragmentation and loss in parkin-expressing 293T cells treated with CCCP. (**a**) Epi-fluorescence microscopy of 293T WT cells, transfected with 6xMyc-parkin WT or V380L and treated with different concentrations of CCCP for 24 h. Mitochondria were visualised by co-expressing DsRed2-Mito (red). DAPI was used as a nuclear counterstain (blue). Images show magnifications of areas marked with white dashed boxes in Fig. 3a. Scale bar = 5 µm. (**b**) Confocal microscopy of 293T WT cells co-expressing DsRed2-Mito and 6xMyc-parkin WT or V380L, and treated with 50 µM CCCP for 24 h. Mitochondrial structures are displayed as greyscale signals on a white background. 'Nuc' indicates approximate location of the cell nucleus. Scale bar = 5 µm

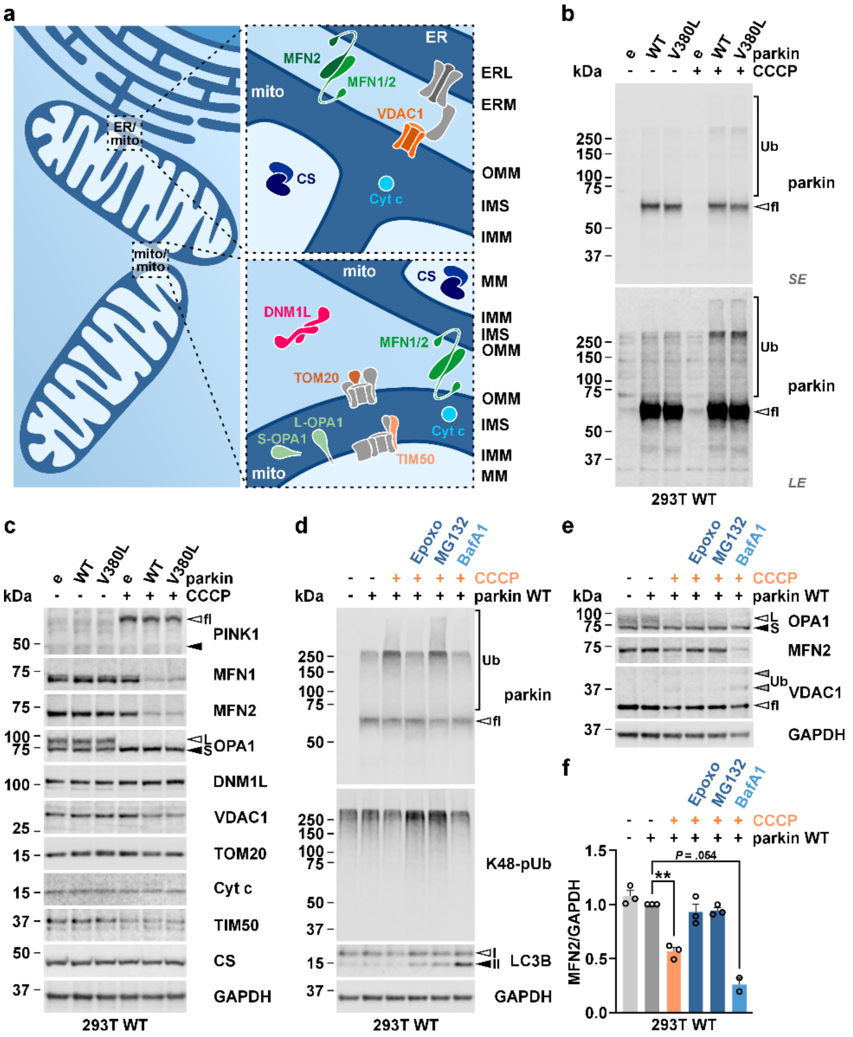

Supplementary Fig. S11 Both parkin variants can promote mitophagy in 293T cells upon CCCP administration. (**a**) Schematic illustration of the endoplasmic reticulum-mitochondria and mitochondria-mitochondria interaction sites and the localization of analysed proteins in context of mitophagy. ER, endoplasmic reticulum; ERL, ER lumen; ERM, ER membrane; mito, mitochondrion; OMM, outer mitochondrial membrane; IMS, intermembrane space; IMM, inner mitochondrial membrane; MM, mitochondrial matrix. (**b**, **c**) Western blotting of 293T WT cells transfected with an empty vector (e), or 6xMyc-parkin WT or V380L, and treated with 12.5 µM CCCP 24 h prior to harvest. Membranes were probed with antibodies against parkin, PINK1, mitofusin-1 (MFN1), MFN2, optic atrophy protein 1 (OPA1), dynamin 1-like protein (DNM1L), VDAC1, translocase of the outer mitochondrial membrane 20 (TOM20), cytochrome c (Cyt c), translocase of the inner mitochondrial membrane 50 (TIM50), and citrate synthase (CS). The data demonstrated that mitophagy induction in parkin-overexpressing cells using CCCP leads to parkin-dependent and independent alterations of several mitochondrial protein markers. GAPDH served as loading control. (**d, e**) Western blotting of 293T WT cells transfected with an empty vector (e) or 6xMyc-parkin WT, and treated with 12.5 µM CCCP in combination with proteasome inhibitors epoxomicin (Epoxo) or MG132, or with autophagy inhibitor bafilomycin A1 (BafA1) 24 h prior to harvest. Membranes were probed with antibodies against parkin, OPA1, MFN2, or VDAC1. The analysis substantiates that MFN2 degradation is a proteasome-dependent mechanism. Successful treatment with Epoxo, MG132, or BafA1 was monitored by detecting K48-polyubiquitin chains (K48-pUb) and LC3B-II, respectively. White arrowheads indicate full-length (fl)/long (L) forms of parkin, PINK1 or OPA1. Black arrowheads show cleaved/short (S) forms of PINK1 and OPA1 as well as LC3B-II (II). Brackets mark ubiquitinated (Ub) forms of parkin. *SE*, short exposure, *LE*, long exposure. (**f**) Densitometric quantification of MFN2 levels upon CCCP treatment and inhibition of proteasomal or autophagosomal degradation, normalised to GAPDH and, within each replicate, to the parkin WT/-CCCP control. n = 2-3. Bars represent mean + s.e.m. One sample t-test; $^{**}P \leq 0.01$

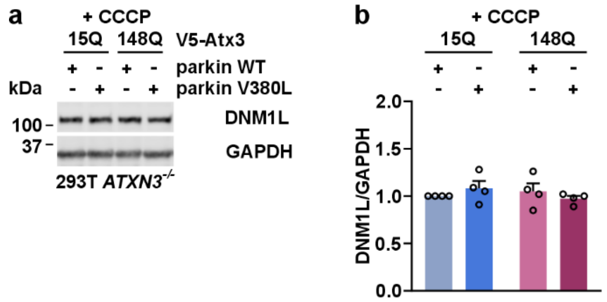

Supplementary Figure S12 No differences in DNM1L levels upon mitophagy induction in cells co-expressing parkin and ataxin-3 variants. (**a**) Western blotting of 293T *ATXN3*$^{-/-}$ cells co-expressing V5-Atx3 15Q or 148Q and 6xMyc-parkin WT or V380L, treated with 12.5 µM CCCP 24 h prior harvest. The membrane was probed with an antibody against DNM1L. No changes are detectable when co-expressing parkin WT or V380L and ataxin-3 15Q or 148Q upon mitophagy induction. GAPDH served as loading control. (**b**) Densitometric quantification of DNM1L levels upon CCCP treatment, normalised to GAPDH. Within each experimental replicate, values were additionally normalised to the control Atx3 15Q/parkin WT/+CCCP. n = 4. Bars represent mean + s.e.m.

Supplementary Tables

Supplementary Table S1 Overview of MJD patient cohorts analysed in this study.

Cohort	EUROSCA [a]	EuSAge [b]	Montreal [c]	All cohorts combined
**Patients, *n***	517	76	318	911
**Evaluable samples, *n***	436	54	318	808
**Independent samples, *n***	277	23	136	436
**Family members, *n* (mean)**	1.57	2.35	2.30	1.85
**Family members, *n* (median) [range]**	6 [1-12]	4 [1-7]	7 [1-18]	7.5 [1-18]
**CAG repeats, *n* (mean) ± s.e.m.**	71.5 ± 0.2	65.4 ± 0.6	71.9 ± 0.2	71.2 ± 0.2
**CAG repeats, *n* (median) [range]**	72 [58-82]	66 [56-76]	72 [62-82]	72 [56-82]
**AAO, years (mean) ± s.e.m.**	39.4 ± 0.6	45.4 ± 1.8	39.7 ± 0.7	39.9 ± 0.4
**AAO, years (median) [range]**	40 [10-78]	47.5 [20-68]	40 [10-70]	40 [10-78]
**rs1801582 G/G, *n* [percent]**	277 [63.5]	43 [79.6]	216 [67.9]	536 [66.3]
**rs1801582 G/C, *n* [percent]**	147 [33.7]	10 [18.5]	90 [28.3]	247 [30.6]
**rs1801582 C/C, *n* [percent]**	12 [2.8]	1 [1.9]	12 [3.8]	25 [3.1]

The table shows all relevant information on sub-cohorts and the combined cohort. The number of patients as well as the number of evaluable and independent samples are presented. Additionally, the means and medians (with the range) of the numbers of family members are listed. MJD families included in this study were not restricted to parents and their children. Samples that were not evaluated were excluded based on the following criteria: no successful genotyping possible, missing values for CAG repeats or AAO, or missing family information. One sample in the EUROSCA group had a CAG repeat count in the non-pathological range (*nCAG* = 50) and was also excluded. To account for multiple patients within one family, we applied a family factor and considered the whole family as one independent sample only. Means (± s.e.m.) and medians (with range) of CAG repeat numbers and age at onset (AAO), as well as the distribution (numbers of individuals and percentages) of the different genotypes for *PRKN* SNP rs1801582 are listed. [a] The EUROSCA samples were collected within the European integrated project on spinocerebellar ataxias (EUROSCA) consortium. [b] EuSAge samples were collected within the European-South American collaborative project to identify and characterize age-related genetic modifiers and biomarkers of neurodegenerative processes in SCA3/MJD (EuSAge). Due to the smaller sample size, the mean CAG repeats and AAO slightly differ in the EuSAge cohort but are in the same range. [c] Samples from the Montreal cohort, consisting of MJD patients from Portugal and Canada, were collected by Jorge Sequeiros and Guy A. Rouleau, and genotyped by Sandra Martins.

Supplementary Table S2 Primers and probes employed for PCR-based amplification, sequencing, and high-resolution melting analysis of selected missense SNP variants in the *PRKN* gene.

**SNP**	**Orientation**	**Sequence (5‘-3‘)**
rs1801474,<br>gnomAD ID: 6-162201165-C-T,<br>*PRKN* exon 4,<br>c.601G>A<br>(p.S167N)	forward primer	AAAGGCCCCTGTCAAAGAGT
	reverse primer	TCATTTTCCTGGCAGTCTCA
	forward probe (pm)	AGGGTACAGTGCAGCACCTGCAGGCAG-**P**
rs1801582,<br>gnomAD ID: 6-161386823-C-G,<br>*PRKN* exon 10,<br>c.1239G>C<br>(p.V380L)	forward primer	CTGCCGGGAATGTAAAGAAG
	reverse primer	GGAACTCTCCATGACCTCCA
	forward probe (mm)	GGGAGTGCAGTGCCCTATTTGAAGCCTCA-**P**
rs1801334,<br>gnomAD ID: 6-161360193-C-T,<br>*PRKN* exon 11,<br>c.1281G>A<br>(p.D394N)	forward primer	CCGACGTACAGGGAACATAAA
	reverse primer	CTGCTCAGCACAGACTCACC
	forward probe (pm)	ACAGAGTCGATGAAAGAGCCGCCG-**P**

-P marks a phosphate modification at the probe’s 3’ end. mm = mismatch; pm = perfect match. Underlined bases mark positions of SNPs within the sequence. All gnomAD IDs refer to the GRCh38/hg38 genome build and gnomAD version v4.1.0.

Supplementary Table S3 *PRKN* SNP-specific reaction mixtures for the PCR-based amplification of DNA samples.

	**rs1801582 (exon 10)**	**rs1801474 (exon 4)**<br>**rs1801334 (exon 11)**
OneTaq buffer<br>(New England Biolabs, Frankfurt, Germany)	4.0 µl (5×)	2.0 µl (10×)
dNTP (2 mM)	1.6 µl	1.6 µl
Forward Primer (10 pmol/µl)	0.8 µl	0.8 µl
Reverse Primer (10 pmol/µl)	0.8 µl	0.8 µl
OneTaq DNA Polymerase<br>(New England Biolabs, Frankfurt, Germany)	0.125 µl	0.2 µl
Betaine	4.0 µl	-
H_2O (Ampuwa)<br>(Fresenius Kabi, Bad Homburg Germany)	7.7 µl	10.6 µl
DNA	1.0 µl	4.0 µl
**Total**	**20.0 µl**	**20.0 µl**

Supplementary Table S4 *PRKN* SNP-specific touchdown program for PCR-based amplification of sample DNA.

**Step**	**Cycles**	**Time**	**Temperature (°C)**
Initial denaturation	1	1 min (4 min)	94
Denaturation	10	1 min (15 s)	94
Annealing		1 min (30 s)	65 (-1/cycle)
Elongation		1 min (30 s)	68 (72)
Denaturation	25 (30)	1 min (15 s)	94
Annealing		1 min (30 s)	55
Elongation		1 min (30 s)	68 (72)
Final elongation	1	6 min	68 (72)

Table shows parameters for SNP rs1801582. Program adjustments specific for SNPs rs1801474 (exon 4) and rs1801334 (exon 11) are additionally shown in brackets.

Supplementary Table S5 Reaction mixtures used in high-resolution melting analysis for all examined SNPs in *PRKN*.

	**rs1801582 (exon 10)**	**rs1801474 (exon 4)**<br>**rs1801334 (exon 11)**
High-Resolution Melting Master Mix (Roche Diagnostics)	5.0 µl	10.0 µl
Primer mix (forward:reverse)	1 µM:10 µM	4 µM:40 µM
Unlabelled probe	0.4 µM	0.5 µM
$MgCl_2$	2.5 mM	2.5 mM
DMSO	0.4 µl	-
DNA	1.0 µl	x
H_2O	1.6 µl	ad 20.0 µl
**Total**	**10.0 µl**	**20.0 µl**

Supplementary Table S6 Primary antibodies for western blot analysis.

**Antibody/ target**	**Host**	**Dilution**	**Clone/ catalogue no.**	**Vendor/ source**
β-actin	mouse	1:5,000	AC-15/A5441	Sigma-Aldrich
Ataxin-3	mouse	1:5,000	1H9/MAB5360	Merck
Ataxin-3 (isoform 2-specific)	rabbit	1:1,000	SA3637	Schmidt *et al*., 1998 [1]
Citrate synthase	rabbit	1:1,000	ab96600	Abcam
DNM1L	rabbit	1:1,000	NB110-55237	Novus Biologicals
GAPDH	mouse	1:2,000	0411/sc-47724	Santa Cruz
GAPDH	rabbit	1:5,000	10494-1-AP	Proteintech
GFP	mouse	1:1,000	sc-9996	Santa Cruz
LC3B	rabbit	1:1,000*	#2775	Cell Signaling
MFN1	mouse	1:1,000	3C9/ab57602	Abcam
MFN2	mouse	1:500	6A8/ab56889	Abcam
OPA1	mouse	1:1,000	18/OPA-1/612606	BD Biosciences
parkin	mouse	1:1,000	PRK8/MAB5512	Merck
parkin	rabbit	1:1,000*	#2132	Cell Signaling
PARP1	rabbit	1:2,000	13371-1-AP	Proteintech
PINK1	rabbit	1:250	23274-1-AP	Proteintech
α-spectrin	mouse	1:1,000	AA6/MAB1622	Merck
TIM50	rabbit	1:500*	#62317	Cell Signaling
TOM20	mouse	1:200	F-10/sc-17764	Santa Cruz
ubiquitin (K48-linkage specific)	rabbit	1:1,000*	D9D5/#8081	Cell Signaling
V5-tag	mouse	1:1,000	SV5-Pk1/R960-25	Thermo Fisher
VDAC1	mouse	1:200	sc-390996	Santa Cruz
VDAC1	rabbit	1:500	55259-1-AP	Proteintech

For western blot analysis, all antibodies were diluted in 1xTBS with 0.1% (v/v) Tween-20 and 0.02% NaN_3. *Antibody dilutions were supplemented with 5% (w/v) bovine serum albumin.

Supplementary Table S7 Comparison of genotype *PRKN* SNP rs1801582 distribution in MJD cohorts against expected distribution based on the genetic ancestry groups.

Cohort		*PRKN* rs1801582 genotype			χ^2 test, *P*
		G/G	G/C	C/C	
**EUROSCA & Montreal**	Observed	493	237	24	0.204
	Expected [a]	517	215	22	
**EuSAge**	Observed	43	10	1	0.296
	Expected [b]	38	15	1	
**All cohorts combined**	Observed	536	247	25	0.399
	Expected [c]	554	230	24	

Expected genotype occurrence was calculated based on genotyping data for rs1801582 retrieved from the 1000 Genomes Study (https://www.ncbi.nlm.nih.gov/bioproject/PRJEB6930), with minor allele frequencies of [a] G = 0.1730 for European population, [b] G = 0.1640 for the American population, and [c] G = 0.1721 for the global population. Chi-square (χ^2) tests were performed to detect differences in the respective genotype distribution.

Supplementary Table S8 Analysis of differences in CAG repeat numbers and age at onset between *PRKN* SNP rs1801582 genotypes in MJD sub-cohorts and their combinations.

Cohort	Kruskal-Wallis test, *P* (CAG)	Kruskal-Wallis test, *P* (AAO)
**EUROSCA**	0.550	0.294
**EuSAge**	0.088	0.117
**Montreal**	0.075	0.109
**EUROSCA & EuSAge**	0.366	0.194
**EUROSCA & Montreal**	0.461	0.07
**All cohorts combined**	0.213	**0.035**

Differences in the CAG repeat numbers and age at onset (AAO) between genotypes were analysed using a two-tailed Kruskal-Wallis test, after applying a family factor to account for related samples. Significant *P*-values are highlighted in bold.

Supplementary Table S9 Multivariate linear regression analysis with additive and interactive models of MJD sub-cohorts and their combinations.

	**CAG repeats**		**CAG repeats and SNP, additive model (SNP)**			**CAG repeats and SNP, interactive model (CAG*SNP)**		
**Cohorts**	r^2	P	r^2	Δr^2	P	r^2	Δr^2	P
**EUROSCA**	0.478	**< 0.0001**	0.477	-0.001	0.545	0.481	0.003	0.057
**EuSAge**	0.587	**< 0.0001**	0.587	0.000	0.342	0.588	0.001	0.271
**Montreal**	0.438	**< 0.0001**	0.434	-0.004	0.455	0.443	0.005	0.184
**EUROSCA & EuSAge**	0.472	**< 0.0001**	0.471	-0.001	0.643	0.476	0.004	**0.044**
**EUROSCA & Montreal**	0.508	**< 0.0001**	0.508	0.000	0.183	0.510	0.002	0.073
**All cohorts combined**	0.497	**< 0.0001**	0.498	0.001	0.240	0.503	0.006	**0.043**

Models with the influence of the *ATXN3* CAG repeats alone, as well as the influence with an inclusion of the *PRKN* SNP rs1801582 were examined in an additive and an interactive model. A family factor was applied to account for related samples. r^2, coefficient of determination. Significant P-values are highlighted in bold.

Supplementary references

1. Schmidt T, Landwehrmeyer GB, Schmitt I, Trottier Y, Auburger G, Laccone F, et al. An isoform of ataxin-3 accumulates in the nucleus of neuronal cells in affected brain regions of SCA3 patients. Brain Pathol. 1998;8(4):669-79. doi: 10.1111/j.1750-3639.1998.tb00193.x.

Supplementary data

The EUROSCA Network collaborators and their affiliations

Peter Bauer[1,2,3], José Berciano[4], Sylvia Boesch[5], Alexis Brice[6,7], Alexandra Durr[6,7], Sylvie Forlani[6], Paola Giunti[8], Heike Jacobi[9], Thomas Klockgether[10], Bela Melegh[11], Massimo Pandolfo[12], Olaf Riess[1], Tanja Schmitz-Hübsch[13,14,15,16], Ludger Schöls[17], Jörg B. Schulz[18], Giovanni Stevanin[6,19], Sandra Szymanski[20], Sophie Tezenas du Montcel[6], Dagmar Timmann[21], Bart P. C. van de Warrenburg[22]

[1] Institute of Medical Genetics and Applied Genomics, University of Tübingen, Tübingen, Germany
[2] Centogene GmbH, 18055 Rostock, Germany
[3] Clinic for Internal Medicine, Department of Hematology, Oncology, Palliative Medicine, University Medicine Rostock, 18057 Rostock, Germany
[4] Servicio Cántabro de Salud Santander, University Hospital Marqués de Valdecilla (IDIVAL), University of Cantabria (UC), Santander, Spain
[5] Department of Neurology, Medical University Innsbruck, Innsbruck, Austria
[6] Sorbonne Université, Institut du Cerveau - Paris Brain Institute - ICM, Inserm, CNRS, APHP, University Hospital Pitié-Salpêtrière, Paris, France
[7] AP-HP, National Reference Center for Rare Diseases 'Neurogenetics', Pitié-Salpêtrière University Hospital, Paris, France
[8] Department of Molecular Neuroscience, Institute of Neurology, University College London, London, United Kingdom
[9] Department of Neurology, University Hospital Heidelberg, Heidelberg, Germany
[10] German Center for Neurodegenerative Diseases (DZNE), Bonn, Germany; Department of Neurology, University Hospital of Bonn, Bonn, Germany
[11] Department of Medical Genetics, and Szentagothai Research Center, University of Pécs, Pécs, Hungary; Department of Neurology, Zala County Hospital, Zalaegerszeg, Hungary
[12] Université Libre de Bruxelles (ULB), Neurology Service, ULB Hôpital Erasme, ULB Laboratory of Experimental Neurology, Brussels, Belgium
[13] Experimental and Clinical Research Center, a cooperation between the Max Delbrück Center for Molecular Medicine in the Helmholtz Association and Charité Universitätsmedizin, Berlin, Germany
[14] Experimental and Clinical Research Center, Charité-Universitätsmedizin Berlin, corporate member of Freie Universität Berlin and Humboldt-Universität zu Berlin, Berlin, Germany
[15] Max Delbrück Center for Molecular Medicine in the Helmholtz Association, Berlin, Germany
[16] Charité - Universitätsmedizin Berlin, corporate member of Freie Universität Berlin, Humboldt-Universität zu Berlin, and Berlin Institute of Health, NeuroCure Clinical Research Center, Berlin, Germany
[17] Department of Neurodegeneration and Hertie-Institute for Clinical Brain Research, University of Tübingen, Tübingen, Germany; German Center for Neurodegenerative Diseases (DZNE), Tübingen, Germany
[18] Department of Neurology, RWTH Aachen University, Aachen, Germany; JARA-Translational Brain Medicine, Aachen-Jülich, Aachen, Germany
[19] Université de Bordeaux - CNRS-Centre National de la Recherche Scientifique, INCIA-Institut de Neurosciences Cognitives et Intégratives d'Aquitaine, UMR 5287, Bordeaux, France
[20] Neurology Bochum, Ruhr University Bochum, Bochum, Germany
[21] Department of Neurology, Essen University Hospital, University of Duisburg-Essen, Essen, Germany
[22] Department of Neurology, Donders Institute for Brain, Cognition and Behaviour, Radboud University Medical Center, Nijmegen, Netherlands.
